# *Scn2a* haploinsufficient mice display a spectrum of phenotypes affecting anxiety, sociability, memory flexibility and ampakine CX516 rescues their hyperactivity

**DOI:** 10.1186/s13229-019-0265-5

**Published:** 2019-03-28

**Authors:** Tetsuya Tatsukawa, Matthieu Raveau, Ikuo Ogiwara, Satoko Hattori, Hiroyuki Miyamoto, Emi Mazaki, Shigeyoshi Itohara, Tsuyoshi Miyakawa, Mauricio Montal, Kazuhiro Yamakawa

**Affiliations:** 1 Laboratory for Neurogenetics, RIKEN Center for Brain Science, Wako, Saitama 351-0198 Japan; 2 Department of Physiology, Nippon Medical School, Bunkyo-ku, Tokyo, 113-8602 Japan; 3 Division of Systems Medical Science, Institute for Comprehensive Medical Science, Fujita Health University, Toyoake-shi, Aichi 470-1192 Japan; 4 Laboratory for Behavioral Genetics, RIKEN Center for Brain Science, Wako, Saitama 351-0198 Japan; 5 FIRST, Japan Science and Technology Agency, Kawaguchi, Saitama 332-0012 Japan; 6 Section of Neurobiology, Division of Biological Sciences, University of California San Diego, La Jolla, CA 92093 USA

**Keywords:** *Scn2a*, Autism, Schizophrenia, AMPA receptor

## Abstract

**Background:**

Mutations of the *SCN2A* gene encoding a voltage-gated sodium channel alpha-II subunit Nav1.2 are associated with neurological disorders such as epilepsy, autism spectrum disorders, intellectual disability, and schizophrenia. However, causal relationships and pathogenic mechanisms underlying these neurological defects, especially social and psychiatric features, remain to be elucidated.

**Methods:**

We investigated the behavior of mice with a conventional or conditional deletion of *Scn2a* in a comprehensive test battery including open field, elevated plus maze, light-dark box, three chambers, social dominance tube, resident-intruder, ultrasonic vocalization, and fear conditioning tests. We further monitored the effects of the positive allosteric modulator of AMPA receptors CX516 on these model mice.

**Results:**

Conventional heterozygous *Scn2a* knockout mice (*Scn2a*^KO/+^) displayed novelty-induced exploratory hyperactivity and increased rearing. The increased vertical activity was reproduced by heterozygous inactivation of *Scn2a* in dorsal-telencephalic excitatory neurons but not in inhibitory neurons. Moreover, these phenotypes were rescued by treating *Scn2a*^KO/+^ mice with CX516. Additionally, *Scn2a*^KO/+^ mice displayed mild social behavior impairment, enhanced fear conditioning, and deficient fear extinction. Neuronal activity was intensified in the medial prefrontal cortex of *Scn2a*^KO/+^ mice, with an increase in the gamma band.

**Conclusions:**

*Scn2a*^KO/+^ mice exhibit a spectrum of phenotypes commonly observed in models of schizophrenia and autism spectrum disorder. Treatment with the CX516 ampakine, which ameliorates hyperactivity in these mice, could be a potential therapeutic strategy to rescue some of the disease phenotypes.

**Electronic supplementary material:**

The online version of this article (10.1186/s13229-019-0265-5) contains supplementary material, which is available to authorized users.

## Background

Approximately 5–6% of children are born with developmental disorders [1]. Over the past few years, large-scale studies have identified a large number of mutated genes, among which one of the most recurrent is *SCN2A* [2]. *SCN2A* encodes Nav1.2, a member of the voltage-gated sodium channels (VGSCs) family, essential players of the electrogenesis in excitable cells as they ensure the generation and propagation of action potentials. The first reported case of *SCN2A* mutations was found in a patient with atypical generalized epilepsy with febrile seizures plus (GEFS+) thought to be caused by a slower inactivation of the channel [3]. Thereafter, several reports described missense mutations associated with benign familial neonatal-infantile seizures (BFNIS) [4–6] and multiple de novo missense mutations in epileptic encephalopathies (EE) [7–11].

The symptoms induced by a deficiency of *SCN2A* are however not limited to epilepsies. We previously identified the first de novo nonsense mutation in a patient affected by an association of intractable seizures, autism spectrum disorder (ASD), and intellectual disability (ID) [12]. Since then, numerous case reports described de novo loss-of-function mutations in patients with ASD [13, 14], ID [15, 16], and more recently schizophrenia (SCZ) [17, 18]. Additionally, recent large-scale whole exome sequencing studies identified de novo mutations in *SCN2A* as the most frequent in patients with ASD, SCZ, or ID [19–23]. Considering the various mutations and the consequent phenotypic spectra, it is now recognized that *SCN2A* gain of (increased or accelerated but not toxic) function mutations lead to early infantile-onset severe EEs such as Ohtahara syndrome, while loss-of-function mutations cause ASD, ID, or SCZ with later-onset, milder or even without epilepsies [24–26].

Nav1.2 is dominantly expressed in the axon initial segment of glutamatergic neurons, in particular in neocortical pyramidal cells [6, 27–29] as well as in hippocampal dentate and cerebellar granule cells [30, 31]. Recent reports however also identified Nav1.2-positive medium spiny neurons (MSNs), suggesting that GABAergic inhibitory neurons also express this Nav1.2 channel [32]. Our recent work confirmed this proposal given that a subset of caudal ganglionic eminence-derived inhibitory neurons (mainly vasoactive intestinal peptide (VIP)-positive or reelin-positive/somatostatin-negative) within the hippocampus and neocortex also express Nav1.2 [29]. The contribution of these different Nav1.2 populations of neurons and the mechanisms leading to the various phenotypes observed in cases of mutations in the *SCN2A* gene however remain to be elucidated.

In *Scn2a* haploinsufficient mice, we recently reported impaired excitatory transmission-dependent epileptic phenotypes mimicking later-onset milder epilepsies in ASD patients [33] and impairments in learning/memory and replay of hippocampal place cells [34]. However, other aspects of the behavioral phenotype of the *Scn2a*-haplodeficient mice have not been fully investigated yet.

Here, we undertook a comprehensive, in-depth investigation of the behavioral abnormalities of *Scn2a* conventional and conditional knockout (KO) mice and pharmacologically challenged the glutamate signaling system as a potential mechanism leading to hyperactivity in these mice. Our results show that *Scn2a* haploinsufficiency in mice lead to a spectrum of behavioral phenotypes that have been observed in common models of SCZ and ASD.

## Materials and methods

### Animals and experimental conditions

All animal breeding and experimental procedures were performed in accordance with the guidelines of the Animal Experiment Committee of RIKEN Center for Brain Science and Fujita Health University. Animals were maintained on 12 h light/dark cycle with ad libitum access to food and water. All tests involved male adult mice and were conducted in the light phase of the light/dark cycle, except for the home-cage monitoring that covered both light and dark phases. Mice were given a minimum of 30-min habituation to the experimental room before the start of the tests. Experiments were conducted in a blinded manner. The composition of cohorts and age of the mice for every experiment are summarized in the Additional file 1: Table S1. For social behavior-related tasks, housing condition was homogenized across the different testing batches to prevent a bias as follows: for batches 1, 2, and 4, all mice were housed in groups of two to five individuals per cage until the novel open field test and were then individually housed. For batch 3, mice were individually housed for 3 weeks prior to test to establish a home-cage territory. For batch 5, mice were paired with an age-matched female for 2 weeks prior to test to establish a home-cage territory. For batch 6, mice were individually housed for 2 weeks prior to test.

*Scn2a* conventional (*Scn2a*^KO/+^) [35] and conditional (*Scn2a*^fl/+^) [33] KO, *Empty spiracles homolog 1* (*Emx1*)-Cre knock-in [36], and *Vesicular GABA transporter* (*Vgat*)-Cre BAC transgenic [37] lines were described previously and maintained on a congenic C57BL/6J background.

### Open field

Mice, aged 8 weeks, were placed in a 60 × 60 cm square automated open-field homogeneously illuminated at 70 lx and allowed to freely explore for 30 min. Data was acquired and analyzed using manufacturer’s tracking software (TimeOFCR4; O’Hara & Co. Tokyo, Japan). A second 40 × 40 cm automated open-field illuminated at 100 lx was used to assess exploratory behavior for a longer period of 120 min (Accuscan Instruments, Columbus, OH, USA).

### Elevated-plus maze

The elevated-plus maze consists of two open arms (25 × 5 cm) crossing two enclosed arms of identical dimensions equipped with 15 cm high transparent walls placed 60 cm above the floor. Luminosity was homogeneously set at 70 lx. Male mice, aged 8 to 9 weeks, were placed at the center of the maze, facing one of the open arms and allowed to freely explore for 10 min. Data was acquired and analyzed using manufacturer’s tracking software (TimeEP2; O’Hara & Co. Tokyo, Japan).

### Drug administration

Adult male mice were injected with vehicle (saline, 10 μl/g of body weight) or CX516 (10, 25 or 40 mg/kg, diluted in saline for an injected volume of 10 μl/g of body weight; Sigma-Aldrich # SML1191) intraperitoneally 10 min prior to open field, elevated plus maze, and rotarod tasks.

### Light/dark box

The light (250 lx) and dark (0 lx) boxes were two equal-sized plastic boxes (20 × 20 cm) separated by a central partition plate with a small 3 × 5 cm opening allowing mice to transit from one side to the other. Mice, aged 10 weeks, were placed on the dark side of the apparatus and allowed to freely explore for 10 min. Data was acquired and analyzed using manufacturer’s tracking software (TimeLD4; O’Hara & Co. Tokyo, Japan).

### Three-chambers social interaction

The three-chambers apparatus is a rectangular 43 × 63 cm non-transparent gray Plexiglas box, separated into three equivalent-sized (21 × 43 cm) chambers by transparent Plexiglas plates with small openings allowing mice to freely transit from a chamber to another. One wire quarter-cylinder shaped cage was placed in the corner of each of the side chambers and was used to enclose a 7- to 8-week-old C57BL/6J stranger male mouse. Tested mice, aged 12 ± 1 weeks, were first given a 10-min habituation to the apparatus. A first stranger animal was then placed randomly in a wire cage in one of the side-chambers and the tested mouse given a 10-min free exploration period. In a final step, a second stranger mouse was placed in the wire cage on the opposite side chamber and the tested mouse was given another 10-min exploration period. Data was acquired and analyzed using manufacturer’s tracking software (TimeCSI; O’Hara & Co. Tokyo, Japan).

### Social memory test

Male mice, aged 12 weeks, were individually housed for 3 weeks to establish a home-cage territory. An 8–9-week-old C57BL/6J female was then introduced for four consecutive 1-min-long confrontations spaced by a 10-min interval during which the female was removed from the cage. In each of the consecutive trials, the tested male was exposed to the same female. Ten minutes after the last confrontation, a novel C57BL/6J juvenile female was placed in the cage for 1 min. The total duration of sniffing interactions was monitored for all trials.

### Social dominance tube test

Mice, aged 15 weeks, were first given individually a habituation run through the tube (cylindrical transparent Plexiglas, diameter: 3 cm; length: 30 cm; elevation: 2 cm) in dim light conditions (5 lx). Videos were recorded using an infrared camera. For each round of the test, one *Scn2a*^KO/+^ and one weight-matched WT mouse were introduced at each end of the tube, facing each other and released simultaneously. An animal was considered to have lost the confrontation when both of its posterior limbs touched the table, either from retreating or being pushed out of the tube. Each mouse was given four rounds facing a different randomly chosen opponent each time.

### Resident-intruder

Male mice, aged 9 weeks, were paired with an age-matched female for 2 weeks to establish a home-cage territory. The female was then removed from the cage and a 6–7-week-old Balb/c male was introduced for a 10-min confrontation. Data was acquired for every sniffing, pursuing, and attacking event by the resident mouse toward the intruder male.

### Twenty-four-hour home-cage social interaction monitoring

Two mice of the same genotype, aged 30 ± 1 weeks were placed in clean home cage equipped with an infrared video camera. Animals’ position was tracked over a week using an automated offline ImageJ-based analysis software (ImageHA, designed by Tsuyoshi Miyakawa, available through O’Hara & Co. Tokyo, Japan). Social interaction was evaluated by counting the number of objects detected in each frame: two objects were detected whenever the mice were not in contact, whereas only one object was detected when mice were in contact. Data was recorded for eight consecutive days and the values for days 4 through 6 were averaged.

### Contextual and auditory-cued fear conditioning

For the conditioning phase, 29-week-old mice were place in a transparent cubic box with a stainless steel grid floor in a soundproof chamber with a continuous 50 dB white noise background and homogeneously lit at 210 lx (O’Hara & Co). Mice were given 2 min for habituation and then received two pairs of conditioned stimulus (CS; white noise, 60 dB, 30 s) and unconditioned stimulus (US; electric foot-shock, 0.3 mA, 2 s) spaced by a 1-min gap. After the second CS-US pair, the animal was given 1 min to explore the box and returned to its home cage. For contextual testing (24 h later), mice were placed in the same environmental conditions than for the conditioning phase for 5 min without CS or US. The auditory-cued test was performed 24 h later in an alternative context different in shape (trigonal prism), color (opaque), brightness (50 lx), background white noise (60 dB), and flooring (paper-chips bedding, Alpha-Dri, Shepherd Specialty Products, Kalamazoo, MI, USA). Animal’s behavior was recorded for 2 min to evaluate the nonspecific contextual fear and the subject was then given a single CS (white noise, 65 dB, 2 min) without US. Mice were given a final 1-min period of free exploration. Mice freezing rate was automatically detected using the manufacturer’s software (O’Hara & Co. Tokyo, Japan).

### Conditioned fear extinction

The conditioning phase was performed in the conditioning context described above using a set of mice independent from the group tested in the contextual and auditory-cued fear conditioning task. Mice, aged 13 weeks, were allowed to explore the box for 3 min and received three CS (CS; white noise, 60 dB, 30 s) US (US; electric foot-shock, 0.3 mA, 2 s) pairs spaced by a 1-min interval. The extinction test was performed 24 h later in the auditory-cued test context described above. Each mouse was given a 3-min free exploration period and was then exposed to ten consecutive CS (white noise, 65 dB, 30 s) spaced by a 30-s interval, without US. The probe test was performed 24 h later in the same auditory-cued test chamber. After 3 min of free exploration, mice were given three consecutive CS (white noise, 65 dB, 30 s) spaced by a 30-s interval, without US. Animals’ freezing rate was automatically detected using the manufacturer’s software (O’Hara & Co. Tokyo, Japan).

### Local field potential recordings in freely behaving mice

Adult male mice (3–6 months) were used in these experiments. An antibiotic (ampicillin) was used in surgery. Stainless steel screws (1.1 mm diameter) serving as electrocorticogram (ECoG) electrodes were inserted through the skull in contact with the somatosensory cortex (± 1.5 mm lateral and 1.0 mm posterior from bregma) under 1–1.5% isoflurane anesthesia. A screw electrode in contact with the cerebellum was used as reference. Stainless wire electrodes (100 μm diameter) were inserted in the cervical region of the trapezius muscle to record electromyogram (EMG). To record simultaneous monopolar local field potential (LFP) of different brain regions, beveled-tip insulated stainless wires (200 μm diameter) were stereotaxically implanted contralateral to the ECoG electrode at the following coordinates (in mm; anterior-posterior, medial-lateral, depth from the cortical surface): medial prefrontal cortex (1.9, 0.3, 1.4), caudate-putamen (0.0, 2.4, 2.5), basolateral amygdala (− 1.4, 2.9, 3.7), ventroposterior thalamus (− 1.8, 1.5, 3.2), hippocampus CA1 region (− 2.5, 2.2, 1.1), visual cortex binocular zone (− 3.4, 3.0, 0.4). After at least 1 week of recovery, ECoG and LFPs were recorded for three consecutive days using a 16-channel commutator (Plexon, Dallas, TX). Animal’s behavior was continuously monitored using an infrared camera. LFPs (filtered 0.7–170 Hz, 1 kHz sampling) were recorded using the MAP data acquisition system (Plexon) and analyzed off-line using NeuroExplorer software (Nex Technology, Madison, AL). For power spectrum analysis, a 3-h window of LFP traces during daytime was divided into waking, slow-wave sleep, and rapid eye movement sleep stages, and the power spectra of these segments were calculated using NeuroExplorer (0–100 Hz, 0.390625 Hz bin). To compare absolute LFP powers, power densities corresponding to defined frequency ranges (e.g., gamma 30–80 Hz) were summed. The position of the electrodes was confirmed by sectioning and hematoxylin/eosin staining.

### Statistical analyses

Statistical significance was assessed using paired *t* test, Student’s *t* test, one-way ANOVA, or two-way repeated measures ANOVA with post-hoc Tukey’s or Sidak’s multiple comparisons test using Prism 7 (GraphPad Software, La Jolla, CA, USA). For experiments with unbalanced sample numbers, a REML mixed effect model was applied to process ANOVAs using Prism 7 software. Winning rate in the social dominance tube test was analyzed with a binominal Chi squared test with a 50% expected value. The values shown in graphs and reported in the text are expressed as mean ± SEM with statistical significance set at *p* < 0.05.

## Results

### Heterozygous *Scn2a* KO mice display hyperactivity in novel environments

General health examination of adult *Scn2a*^KO/+^ male mice did not reveal significant changes in gross morphology, body temperature, and sensory response (Additional file 2: Figure S1A-C, Additional file 3). Although muscular strength was unaffected in *Scn2a*^KO/+^ mice (Additional file 2: Figure S1D-E), the rotarod task revealed a significant deficit in locomotor coordination throughout six consecutive learning trials (Additional file 2: Figure S1F).

**Fig. 1:**
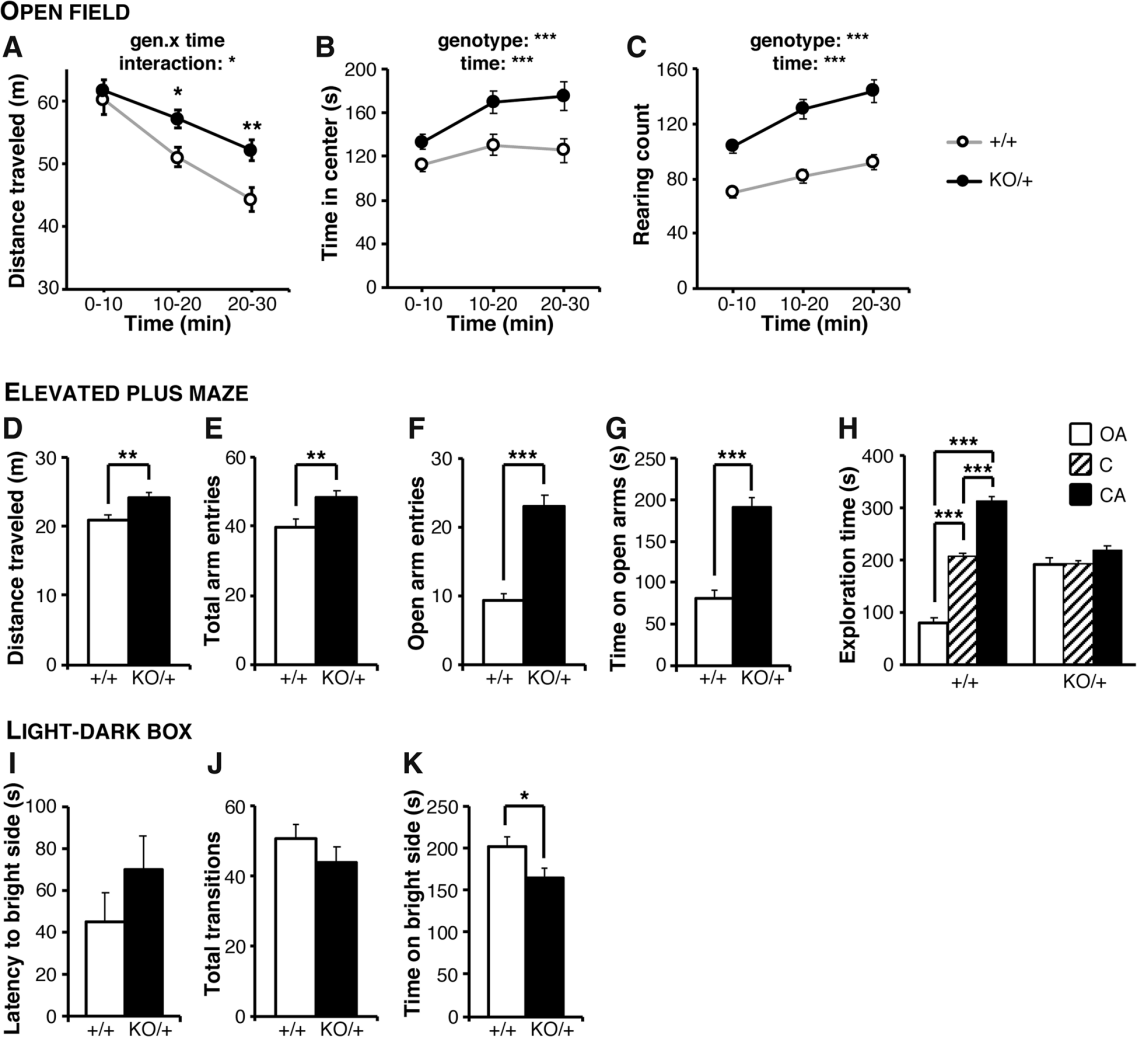
Increased novelty-induced hyperactivity in *Scn2a*^KO/+^ mice. **a** In the open field task, the traveled distance was significantly increased in *Scn2a*^KO/+^ (KO/+) mice in the second and third blocks of 10 min of the test (genotype-time interaction: *F*_2,40_ = 3.137, *p* < 0.05). **b** *Scn2a*^KO/+^ (KO/+) mice spent significantly more time in the center area than their WT (+/+) littermates (genotype-time interaction: *F*_2,40_ = 3.806, NS; time effect: *F*_2,58_ = 8.252, *p* < 0.001; genotype effect: *F*_1,29_ = 18.01, *p* < 0.001). **c** The number of rearing events was also significantly increased in the *Scn2a*^KO/+^ group (genotype-time interaction: *F*_2,40_ = 2.646, NS; time effect: *F*_2,58_ = 38.50, *p* < 0.001; genotype effect: *F*_1,29_ = 36.85, *p* < 0.001). In the elevated-plus maze task, *Scn2a*^KO/+^ mice also showed significant signs of hyperactivity, traveling longer distances (**d**) and visiting the different arms more often (**e**) than their WT littermates. The number of visits to the open arms (**f**) and the time spent in the open arms (**g**) were significantly increased in the *Scn2a*^KO/+^ mice, and whereas WT mice showed a significant preference for the closed arms over the open ones (**h**) this was not the case for *Scn2a*^KO/+^ animals. In the light-dark transition task, the latency to first enter the bright side (**i**) and the total number of side transitions (**j**) were not significantly different between *Scn2a*^KO/+^ and WT mice, but *Scn2a*^KO/+^ animals spent significantly less time on the bright side (**k**). *OA* open arm, *C* center, *CA* closed arm. Open field: WT: *N* = 24, *Scn2a*^KO/+^: *N* = 30; elevated-plus maze: WT: *N* = 24, *Scn2a*^KO/+^: *N* = 29; light-dark box: WT: *N* = 11, *Scn2a*^KO/+^: *N* = 14. Values are expressed as mean ± standard error of the mean. Statistical significance was assessed using two-way repeated measures ANOVA followed by Sidak’s multiple comparison post-hoc test (**a**–**c**), one-way ANOVA (**h**, tested separately for WT and *Scn2a*^KO/+^mice), or Student *t* test (**d**–**g** and **i**–**k**) with significance set at *p < 0.05, **p < 0.01 and ***p < 0.001

We first investigated the spontaneous exploratory behavior using the open field task. Whereas in both *Scn2a*^KO/+^ and WT groups, the distance traveled decayed as a function of time spent exploring the open field; this effect was milder in *Scn2a*^KO/+^ samples and resulted in a significantly longer distance traveled in the second and third blocks of 10 min of the task (Fig. 1a). *Scn2a*^KO/+^ mice spent significantly more time exploring the center of the open field than their WT littermates (Fig. 1b). Additionally, the number of rearing events was drastically larger in the *Scn2a*^KO/+^ group (Fig. 1c). Further investigation showed that the enhanced locomotor activity, increased time spent in the center, and rearing counts in a novel open field lasted for at least the first 60 min of exploration (Additional file 2: Figure S2).

We then used the elevated-plus maze test to further investigate anxiety-like behavior. The distance traveled and total number of arm entries were significantly larger in the *Scn2a*^KO/+^ group (Fig. 1d, e). The number of entries and time spent in the open arms were significantly increased in *Scn2a*^KO/+^ mice (Fig. 1f, g) and while WT mice showed substantial preference for the closed arms, this was not the case for *Scn2a*^KO/+^ animals (Fig. 1h). Although hyperlocomotion might affect the reading of anxiety-related parameters, the drastic increase in entries and time spent in the open arms (145% increase in open arm entries and 136% increase in time spent in the open arms) is unlikely to be caused simply by the modest increase in locomotion (15% increase in total traveled distance and 22% increase in total number of arm entries). Taken together, these results suggest that *Scn2a*^KO/+^ mice are hyperactive and less anxious than their WT littermates when exposed to a new environment. In the light/dark box test, no major differences in the latency to enter the light side and the total number of light/dark transitions were discerned (Fig. 1i, j). Surprisingly, however, *Scn2a*^KO/+^ mice spent significantly less time than their WT littermates on the light side (Fig. 1k). Although this observation would suggest an increase in anxiety, we observed robust signs of decreased anxiety in the open field and elevated-plus maze tasks. It should be noted that the luminosity used in the open field and elevated-plus maze (70 lx) is by far dimmer than in the bright area of the light/dark box task (250 lx). This raises the possibility of a higher sensitivity to intense brightness in *Scn2a*^KO/+^ mice affecting the outcome in this latter task, whereas they show a clear decrease in anxiety in the open field and elevated-plus maze, tasks commonly used to assess spontaneous behavior in rodents.

### Selective deletion of *Scn2a* in dorsal-telencephalic excitatory but not in inhibitory neurons reproduces the increased vertical activity

**Fig. 2:**
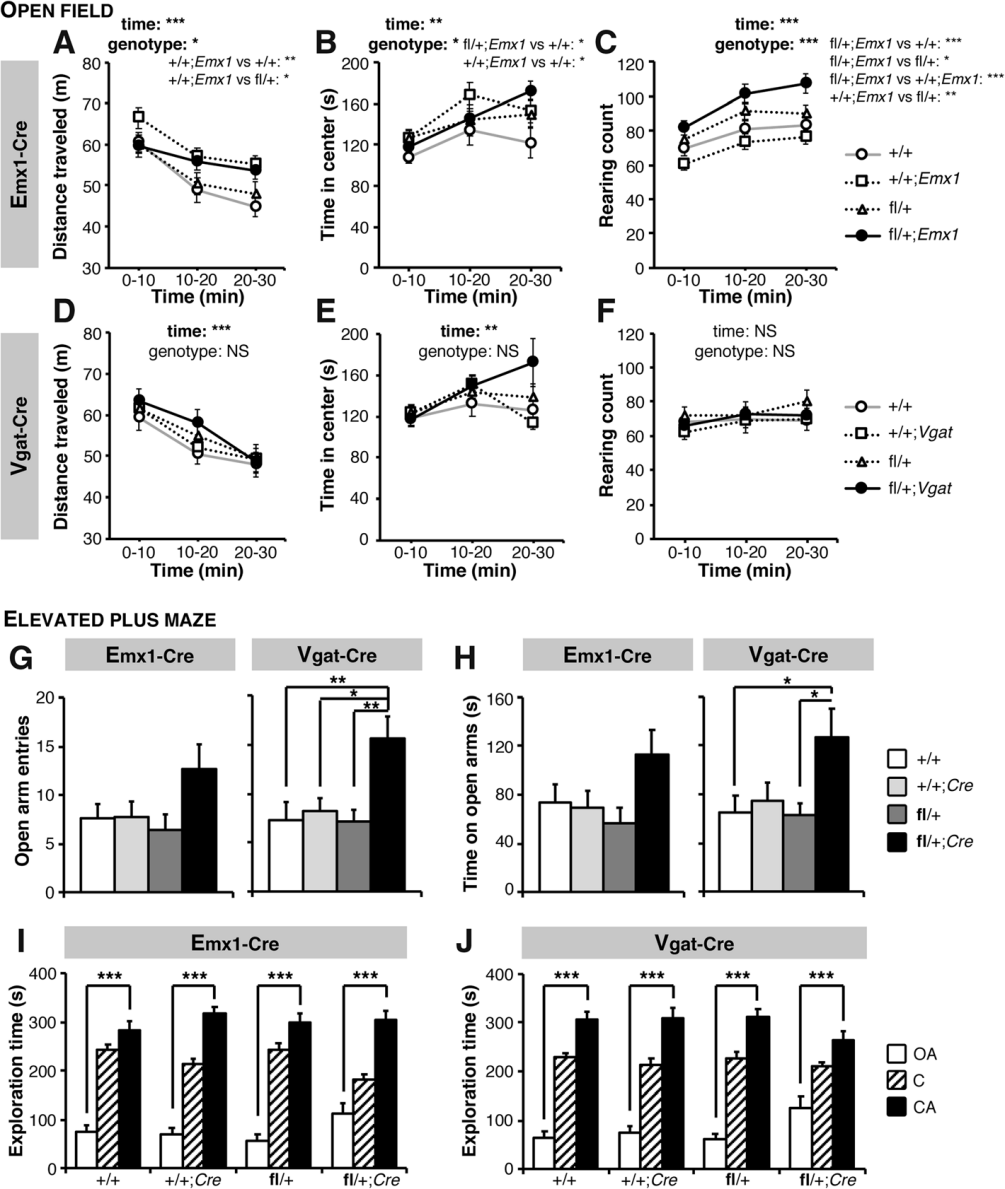
*Emx1*-Cre, but not *Vgat*-Cre-mediated conditional *Scn2a* KO replicates the increased vertical activity in the open field, whereas both affect anxiety-like behavior in the elevated-plus maze. Heterozygous deletion of *Scn2a* in dorsal-telencephalic excitatory neurons led to a mildly, yet not significantly, longer distance traveled (**a**) in the open field in *Scn2a*^fl/+^ (fl/+); *Emx1*-Cre, whereas +/+; *Emx1*-Cre mice traveled significantly longer distances than their +/+ and fl/+ littermates (genotype-time interaction: *F*_3,58_ = 0.372, NS; time effect: *F*_2,62_ = 11.034, *p* < 0.001; genotype effect: *F*_3,13_ = 3.135, *p* < 0.05; post-hoc *p*_+/+;Emx1_vs_+/+_ < 0.01 and *p*_+/+;Emx1_vs_fl/+_ < 0.05). Fl/+; *Emx1*-Cre and +/+; *Emx1*-Cre mice spend significantly more time in the center of the open field than their +/+ littermates (**b**, genotype-time interaction: *F*_6,58_ = 0.784, NS; time effect: *F*_2,62_ = 7.046, *p* < 0.01; genotype effect: *F*_3,13_ = 2.797, *p* < 0.05; post-hoc *p*_fl/+;Emx1_vs_+/+_ < 0.05 and *p*_+/+;Emx1_vs_+/+_ < 0.05). The number of rearing (**c**) was also significantly increased in fl/+; *Emx1*-Cre mice compared to their +/+, fl/+ and fl/+; *Emx1*-Cre littermates (genotype-time interaction: *F*_6,58_ = 0.784, NS; time effect: *F*_2,62_ = 7.046, *p* < 0.01; genotype effect: *F*_3,13_ = 2.797, *p* < 0.05; post-hoc *p*_fl/+;Emx1_vs_+/+_ < 0.001, *p*_fl/+;Emx1_vs_fl/+_ < 0.05, *p*_fl/+;Emx1_vs_+/+;Emx1_ < 0.001 and *p*_+/+;Emx1_vs_fl/+_ < 0.01). In contrast, no significant differences were seen in distance traveled (**d**, genotype-time interaction: *F*_6,33_ = 0.394, NS; time effect: *F*_2,37_ = 22.403, *p* < 0.001; genotype effect: *F*_3,10_ = 1.226, NS), time spent in the center area (**e**, genotype-time interaction: *F*_6,33_ = 2.017, NS; time effect: *F*_2,37_ = 5.019, *p* < 0.01; genotype effect: *F*_3,10_ = 2.055, NS), and rearing count (**f**, genotype-time interaction: *F*_6,33_ = 0.247, NS; time effect: *F*_2,37_ = 1.005, NS; genotype effect: *F*_3,10_ = 1.078, NS) in fl/+; *Vgat*-Cre mice, harboring an inhibitory neuron specific deletion of *Scn2a*, compared to their control +/+, +/+; *Vgat*-Cre and fl/+ mice. In the elevated–plus maze task, fl/+;*Emx1*-Cre mice displayed a tendency and fl/+;*Vgat*-Cre mice a significant increase in number of entries in the open arms (**g**) and time spent in these open arms (**h**) than their respective controls (*Emx1*-Cre: *F*_3,53_ = 2.409, *p* = 0.077 in **g** and *F*_3,53_ = 2.348, *p* = 0.083 in **h**; *Vgat*-Cre: *F*_3,46_ = 5.634, *p* < 0.01 in **g** and *F*_3,46_ = 3.671, *p* < 0.05 in **h**). The significant preference for the closed arms over the open one was however conserved in fl/+;*Emx1*-Cre (**i**) as well as in fl/+;*Vgat*-Cre mice (**j**). *OA* open arm, *C* center, *CA* closed arm. *Emx1*-Cre conditional KO experiment: +/+: *N* = 15, +/+; *Emx1*-Cre: *N* = 15, fl/+: *N* = 11, fl/+; *Emx1*-Cre: *N* = 16. *Vgat*-Cre conditional KO experiment: +/+: *N* = 12, +/+; *Vgat*-Cre: *N* = 13, fl/+: *N* = 13, fl/+;*Vgat*-Cre: *N* = 12. Values are expressed as mean ± standard error of the mean. Statistical significance in **a**–**f** was assessed using two-way repeated measures ANOVA followed, when a main effect of genotype was observed, by a one-way ANOVA and a Tukey’s post-hoc test. Statistical significance in **g**–**j** was assessed using one-way ANOVA across (**g**–**h**) or within (**i**, **j**) groups of genotype. Significance was set at *p < 0.05, **p < 0.01, and ***p < 0.001

Because *Scn2a* is expressed in both excitatory and inhibitory neurons [29], we sought to determine if a critical population of neurons was underlying the hyperactivity and increased rearing. We used conditional KO *Scn2a*^fl/+^/*Emx1*-Cre and *Scn2a*^fl/+^/*Vgat*-Cre mice carrying a selective heterozygous deletion of *Scn2a* in dorsal-telencephalic (e.g., neocortex, hippocampus) excitatory neurons and inhibitory neurons respectively [36, 37]. The effective Cre-loxP-guided recombination in these conditional knockout models has been confirmed in our previous work [33]. *Scn2a*^fl/+^/*Emx1*-Cre mice appeared to travel slightly longer distances than WT mice, but this did not reach the significance level (Fig. 2a). *Scn2a*^fl/+^/*Emx1*-Cre mice spent significantly more time at the center of the open field, but the significant increase in the *Emx1*-Cre group suggests that this genetic modification itself might be interfering (Fig. 2b), as they also showed a significant increase in distance traveled (Fig. 2a). In addition, the rearing count in the *Scn2a*^fl/+^/*Emx1*-Cre group was significantly increased compared to their WT, *Scn2a*^fl/+^, and *Emx1*-Cre littermates (Fig. 2c), mimicking our observations in the *Scn2a*^KO/+^ group (Fig. 1c). In contrast, *Scn2a*^fl/+^/*Vgat*-Cre mice showed no significant alterations in the distance traveled, time spent in the center of the open field, and rearing counts throughout the whole exploration period (Fig. 2d–f). These results suggest that Nav1.2 dysfunction in dorsal-telencephalic excitatory neurons underlies the novelty-induced increase in vertical activity.

We next assessed whether conditional deletion of *Scn2a* in excitatory or inhibitory neurons had an impact on anxiety-like behavior in the elevated-plus maze task. The number of entries and time spent in the open arms tended to be larger in *Scn2a*^fl/+^/*Emx1*-Cre mice and was significantly larger in *Scn2a*^fl/+^/*Vgat*-Cre mice than in their respective controls (Fig. 2g, h), as observed in *Scn2a*^KO/+^ mice (Fig. 1f, g). *Scn2a*^fl/+^/*Emx1*-Cre and *Scn2a*^fl/+^/*Vgat*-Cre mice however showed a significant preference for the closed arms over the open ones (Fig. 2i, j), whereas *Scn2a*^KO/+^ animals failed to show this pattern (Fig. 1h). In this task, the total number of arm entries and total traveled distance were not significantly different across the groups suggesting that the increase in open arm entries and time spent in the open arms are unlikely to be caused by hyperlocomotion (Additional file 2: Figure S3). These observations suggest that Nav1.2 dysfunction in inhibitory cells induces a decrease in anxiety-like behavior in the elevated plus maze, but the combined contribution of both neuron populations appears to be required to observe the stronger phenotype seen in *Scn2a*^KO/+^ mice.

### Treatment with the positive regulator of AMPA receptors CX516 ameliorates hyperactivity in *Scn2a*^KO/+^ mice

**Fig. 3:**
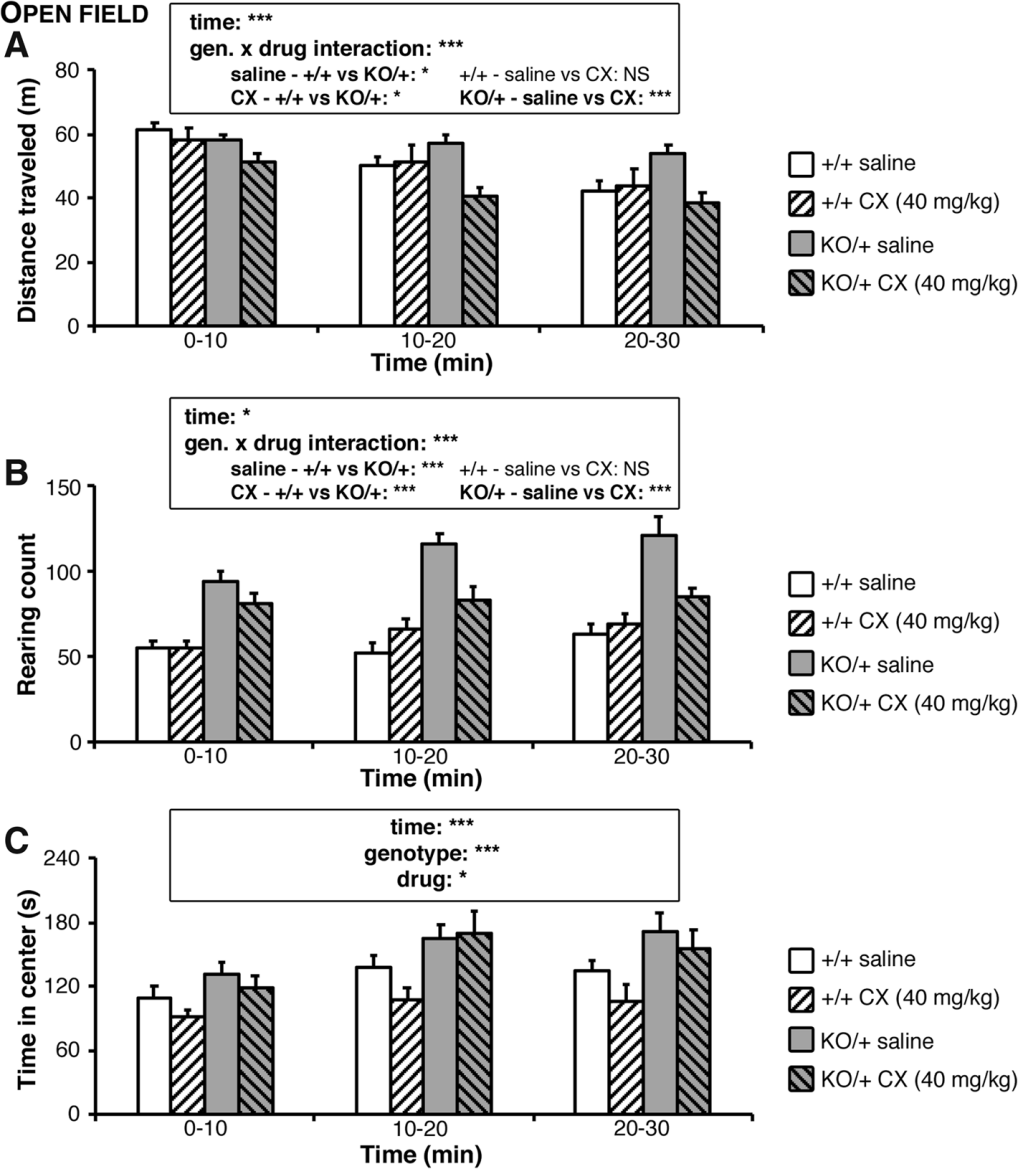
Treatment by AMPA receptor modulator CX516 rescues hyperactivity and increased rearing behavior in *Scn2a*^KO/+^ mice. CX516, a specific AMPA receptor positive allosteric modulator, was intraperitoneally administered 10 min before submitting WT and *Scn2a*^KO/+^ mice to the open field task. **a** The traveled distance decreased significantly over time regardless of the genotype and presence/absence of treatment (time-genotype-treatment interaction: *F*_2,120_ = 1.575, NS; time effect: *F*_2,120_ = 16.195, *p* < 0.001). A significant genotype-treatment interaction was observed, CX516 leading to a significant decrease in traveled distance specifically in the *Scn2a*^KO/+^ group (genotype-treatment interaction: *F*_1,120_ = 12.285, *p* < 0.001; post-hoc WT_saline_vs_CX_: *F*_1,65_ = 0.001, NS; KO_saline_vs_CX_: *F*_1,65_ = 32.406, *p* < 0.001). Saline injected *Scn2a*^KO/+^ mice (KO/+) traveled significantly longer distances than their WT (+/+) littermates whereas CX516-treated *Scn2a*^KO/+^ mice traveled significantly shorter distances (post-hoc saline_WT_vs_KO_: *F*_1,65_ = 4.582, *p* < 0.05; CX_WT_vs_KO_: *F*_1,65_ = 5.428, *p* < 0.05). **b** The rearing count slightly, yet significantly increased over time regardless of the genotype and presence/absence of treatment (time-genotype-treatment interaction: *F*_2,120_ = 2.212, NS; time effect: *F*_2,120_ = 4.646, *p* < 0.05). A significant genotype-treatment interaction was observed, CX516 leading to a significant decrease in rearing count specifically in the *Scn2a*^KO/+^ group (genotype-treatment interaction: *F*_1,120_ = 23.418, *p* < 0.001; post-hoc WT_saline_vs_CX_: *F*_1,65_ = 2.492, NS; KO_saline_vs_CX_: *F*_1,65_ = 21.676, *p* < 0.001). Saline injected *Scn2a*^KO/+^ mice reared significantly more than their WT littermates (post-hoc saline_WT_vs_KO_: *F*_1,65_ = 90.141, *p* < 0.001), and although a significant improvement was seen in the CX516-treated *Scn2a*^KO/+^ mice, their rearing count remaining significantly higher than in controls (CX_WT_vs_KO_: *F*_1,65_ = 17.427, *p* < 0.001). **c** The time spent in the center increased significantly over time, was significantly increased in the *Scn2a*^KO/+^ mice and treatment with CX516 led to a mild yet significant decrease regardless of genotype and time (time-genotype-treatment interaction: *F*_2,120_ = 0.351, NS; time effect: *F*_2,120_ = 7.583, *p* < 0.001; genotype effect: *F*_2,120_ = 24.710, *p* < 0.001; drug effect: *F*_2,120_ = 4.920, *p* < 0.05). The test was conducted using *N* = 11 mice per genotype and per condition (saline and CX516 at 40 mg/kg). Values are expressed as mean ± standard error of the mean. Statistical significance was assessed using three-way ANOVA for time, genotype and treatment effects, followed by one-way ANOVA post-hoc test when a significant interaction was observed (**a**, **b**) with significance set at *p < 0.05, **p < 0.01, and ***p < 0.001

As the enhanced rearing observed in *Scn2a* KO mice was reproduced by a selective *Scn2a* deletion in dorsal-telencephalic excitatory neurons, we hypothesized that acting on the glutamatergic system would help recover phenotypes affecting spontaneous exploration. To challenge this hypothesis, we used intraperitoneal injections of CX516, a specific AMPA receptor positive allosteric modulator, administered 10 min before proceeding to the open field task. Testing this treatment at three different concentrations (10, 25, and 40 mg/kg) did not affect significantly WT animals but appeared to decrease the activity of *Scn2a*^KO/+^ animals with a maximal effect seen at 40 mg/kg (Additional file 2: Figure S4). As observed in non-treated *Scn2a*^KO/+^ mice, saline-injected *Scn2a*^KO/+^ animals traveled significantly longer distances and reared significantly more than their saline-injected wild-type (*Scn2a*^+/+^) littermates (Fig. 3a, b). Although CX516 did not affect significantly wild-type mice, we observed a significant decrease in traveled distance and rearing counts in the *Scn2a*^KO/+^ group especially pronounced in the second and third blocks of 10 min of the task, reaching a level even significantly lower than in the WT groups (Fig. 3a, b). Saline-injected *Scn2a*^KO/+^ mice showed an increase in time spent in the center area in the open field and in the open arms in the elevated-plus maze compared to their saline-injected WT littermates (Fig. 3c, Additional file 2: Figure S5). Treatment with CX516 however did not affect significantly these parameters. Although a significant, non-genotype-specific drug effect was observed in the time spent in the center of the open field, the extent of this effect remained minor in comparison to what was seen in terms of traveled distance and rearing count. As observed in our previous work [38], CX516 injections did not alter animals’ locomotor performance in the rotarod task (Additional file 2: Figure S6). These results thus indicate that the positive regulation of AMPA receptors by an ampakine effectively ameliorates novelty-induced hyperactivity and increased rearing in *Scn2a*^KO/+^ mice without however affecting their low anxiety-like behavior.

### Decreased approach behavior in the resident-intruder’s task and experience-dependent social dominance in *Scn2a*^KO/+^ mice

**Fig. 4:**
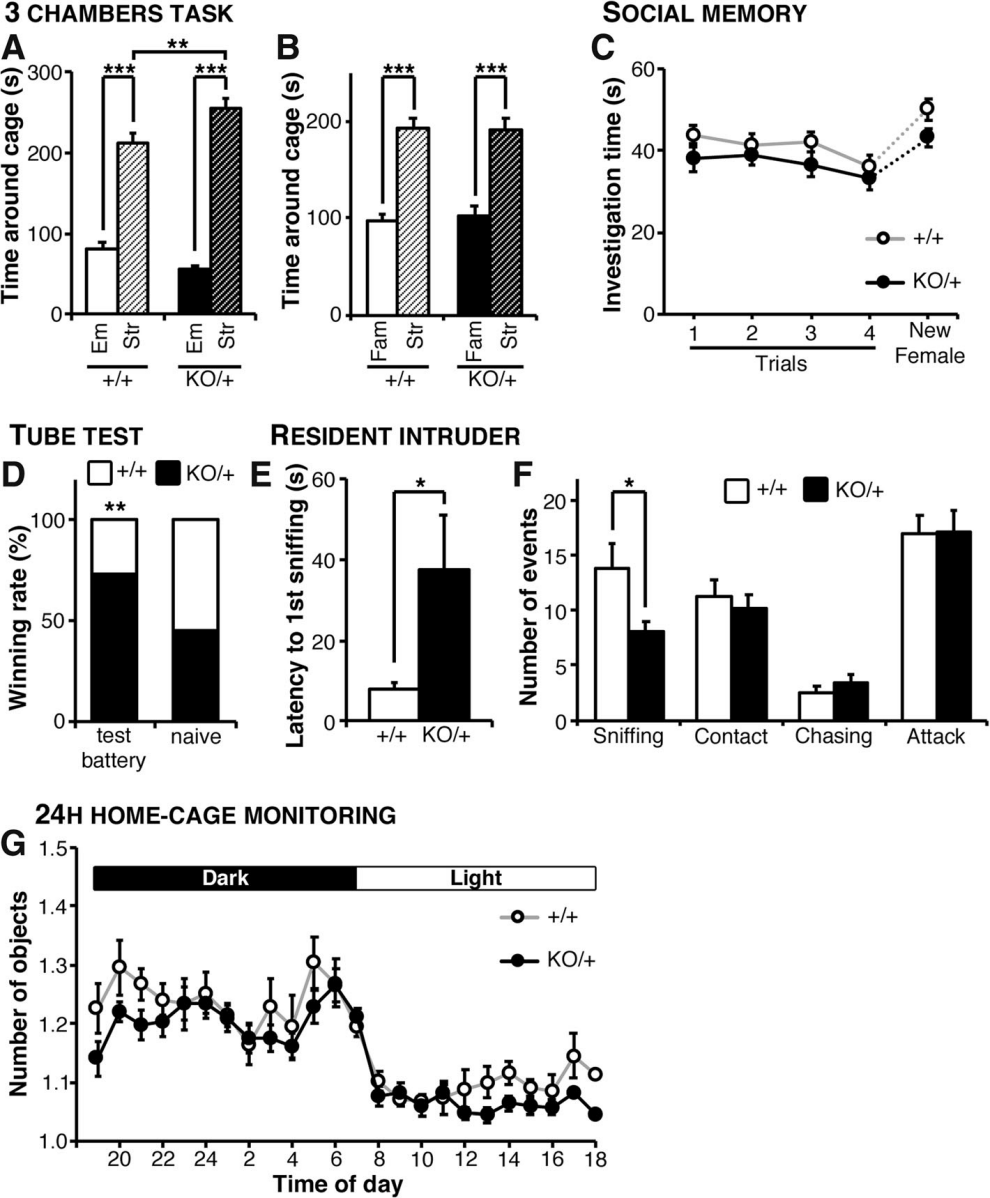
Social memory is conserved but social approach in familiar environment is deficient in *Scn2a* KO mice. **a** In the “sociability” phase of the three-chambers task, *Scn2a*^KO/+^ and WT mice spent significantly more time investigating the stranger mouse than the empty cage, and the time spent on the stranger’s cage was slightly yet significantly longer in the *Scn2a*^KO/+^ group (genotype-side interaction: *F*_1,50_ = 9.524, *p* < 0.01). **b** In the “social memory” phase of the task, mice from both groups showed a significant preference for the novel mouse over the familiar one and no significant genotype effect was observed (genotype-side interaction: *F*_1,50_ = 0.059, NS; side effect: *F*_1,50_ = 52.740, *p* < 0.001; genotype effect: *F*_1,50_ = 0.117, NS). **c** In the direct interaction social memory paradigm (assessed in an unfamiliar environment), animals from both groups gradually habituated to the first intruder. A significant gain of interest was observed upon inserting a new female. No significant differences were however observed between *Scn2a*^KO/+^ and WT mice (genotype-trial interaction: *F*_4,39_ = 0.383, NS; trial effect: *F*_4,44_ = 7.840, *p* < 0.001; genotype effect: *F*_1,11_ = 3.217, NS). **d** In the tube test for social dominance, *Scn2a*^KO/+^ mice that underwent the testing battery won significantly more encounters than their WT littermates (Chi-squared test; *p* < 0.05). The winning rate was however at a random level in naïve *Scn2a*^KO/+^ mice (i.e., that never experienced behavioral tasks). **e** When exposed to a young intruder male in their home cage, the latency of *Scn2a*^KO/+^ mice to the first sniffing contact was significantly longer than in the WT group. **f** The total number of sniffing event was also decreased in the *Scn2a*^KO/+^ group whereas the number of direct contact, chasing and aggression events was comparable between *Scn2a*^KO/+^ and WT animals. **g** Monitoring social behavior for 24 h showed a comparable interaction time in WT-WT and *Scn2a*^KO/+^-*Scn2a*^KO/+^ pairs without significant genotype effect in either the active (nighttime) or inactive (daytime) periods (genotype-time interaction: *F*_23,207_ = 1.026, NS; time effect: *F*_23,207_ = 20.760, *p* < 0.001; genotype effect: *F*_1,9_ = 2.888, NS). *Str* stranger mouse, *Emp* empty cage. Three-chambers: WT: *N* = 23, KO: *N* = 29; direct interaction social memory: WT: *N* = 11, KO: *N* = 12; tube test: WT (test battery): *N* = 13, KO (test battery): *N* = 13, WT (naïve): *N* = 12, KO (naïve): *N* = 12; resident-intruder: WT: *N* = 13, KO: *N* = 13; 24-h monitor home-cage social interaction monitoring WT: *N* = 10 pairs, KO: NO = 10 pairs. Values are expressed as mean ± standard error of the mean. Statistical significance was assessed using two-way ANOVA (using “side” as a within-subject factor, **a**, **b**) or two-way repeated measures ANOVA (**c**, **g**) followed by Sidak’s multiple comparison post-hoc test, chi-square (**d**) or Student *t* test (**e**, **f**) with significance set at *p < 0.05, **p < 0.01, and ***p < 0.001

We next assessed the social performance of *Scn2a*^KO/+^ in the widely used three-chamber test [39, 40]. In the habituation phase, animals from both groups did not show a significant preference for one side chamber over the other (Additional file 2: Figure S7). In the “sociability” phase of the task mice from both groups showed a significant preference for the stranger mouse over the empty side of the maze (Fig. 4a) but *Scn2a*^KO/+^ mice spent slightly yet significantly more time investigating the stranger mouse than their WT littermates. In the “preference for social novelty” phase of the task, *Scn2a*^KO/+^ and WT mice showed significant preference for the novel stranger over the familiar mouse and no significant genotype-effect was observed (Fig. 4b). In the context of the three-chamber task, the sociability and preference for social novelty are thus conserved in *Scn2a*^KO/+^ mice.

We next investigated social memory in a different context using a direct interaction task. The interaction time decreased throughout the four consecutive exposures to the same intruder and increased when a new female on the fifth trial replaced this intruder, without significant differences between *Scn2a*^KO/+^ mice and their WT littermates (Fig. 4c). The time spent investigating the new female appeared shorter in the *Scn2a*^KO/+^ group but this effect did not reach the significance level (paired *t* test; *P* = 0.1044), overall suggesting that social memory was not altered in *Scn2a*^KO/+^ mice.

We then challenged the social hierarchy and dominance of *Scn2a*^KO/+^ mice using the tube test, matching KO mice to randomly selected WT littermates. The winning rate of *Scn2a*^KO/+^ mice was significantly higher than the 50% expected by chance, suggesting a dominant behavior in the mutant group that underwent the behavioral testing battery (Fig. 4d, Chi-squared test; *p* = 0.0105). In contrast, naïve *Scn2a*^KO/+^ without prior experience of behavioral tasks had a winning percentage that did not differ significantly from a random outcome (Chi-squared test; *p* = 0.601). No marked aggressive behavior was observed during the encounters in both experiments. These observations suggest a dominance behavior in *Scn2a*^KO/+^ mice that is likely consequent to previous experience in social and potentially non-social tasks.

Last in the series, we investigated the social behavior of *Scn2a*^KO/+^ mice in their home cage toward either a young intruder male (resident-intruder’s test) or toward a familiar male in a 24-h monitoring paradigm. In the resident-intruder test, the latency to the first sniffing interaction was significantly longer in the *Scn2a*^KO/+^ group suggesting a deficit in social approach toward a stranger male (Fig. 4e). Additionally, the total number of sniffing events was significantly lower in *Scn2a*^KO/+^ mice, whereas the number of nose-to-nose contacts, chasing, and attack events did not differ significantly between the groups (Fig. 4f). In the 24-h interaction test, in which WT-WT or *Scn2a*^KO/+^-*Scn2a*^KO/+^ pairs were monitored, we did not observe significant differences in interaction time in either active or inactive periods (“dark” and “light” respectively, Fig. 4g). Taken together, these results suggest a decrease in approach behavior toward stranger mice in familiar environments, whereas interactions with familiar mice and social interactions in novel environments are not significantly affected in *Scn2a*^KO/+^ mice.

In addition to these physically characterized social interactions in adult mice, we investigated isolation-induced ultrasonic vocalizations (USV) produced by juvenile male mice at postnatal day 6 (P6; Additional file 2: Figure S8, Additional file 3). No significant differences in basic parameters including the number, duration, frequency, frequency modulation (i.e., the difference between the minimum and peak frequencies within each call), and amplitude (dB) of calls were found between *Scn2a*^KO/+^ and their WT littermates (Additional file 2: Figure S8A-F). Classifying all calls produced into the ten main typical categories identified in C57BL/6J mice showed that *Scn2a*^KO/+^ pups produce a repertoire of vocalizations highly similar to their WT littermates (Additional file 2: Figure S8G-H) [41, 42]. Social communication was thus not significantly affected in *Scn2a*^KO/+^ mice.

### *Scn2a*^KO/+^ mice show conflicting abnormal behavior in a set of depression state screening tasks

We next assessed emotional state in *Scn2a*^KO/+^ mice using the two most common tasks used to detect depression-like behavior in rodents: the forced swim test (FST) and the tail suspension test (TST). In the FST, the percentage of immobility increased significantly over the 10-min course of the task in WT and *Scn2a*^KO/+^ mice but this effect was enhanced in the mutant mice (Additional file 2: Figure S9A, Additional file 3), and this effect was still observed, though milder, approximately 24 h later. Consequently, the distance traveled by *Scn2a*^KO/+^ mice on the first day was significantly decreased but unaltered on the second testing day (Additional file 2: Figure S9B). In contrast, *Scn2a*^KO/+^ mice were significantly less immobile than their WT counterparts in the TST (Additional file 2: Figure S9C). Taken together, these results indicate that *Scn2a*^KO/+^ mice have an aberrant behavior divergent in two different tasks commonly used to screen depression state. Although these two parameters are both meant to detect behavioral despair in mice, they involve different pathological mechanisms: reportedly, FST is dopamine-dependent, whereas TST relies on both the serotoninergic and dopaminergic system and this led to discordant observations in the pretreatment of a chemically induced model of negative symptoms with clozapine [43]. This led authors to suggest that the FST is more appropriate to study avolition in models of SCZ.

Deficiencies in startle prepulse inhibition (PPI) have been observed in patients with SCZ and in SCZ model mice [44, 45]. We did not observe significant differences in the startle responses and PPI between *Scn2a*^KO/+^ and WT mice (Additional file 2: Figure S9D-E).

### *Scn2a*^KO/+^ mice show enhanced fear learning and impaired memory extinction

**Fig. 5:**
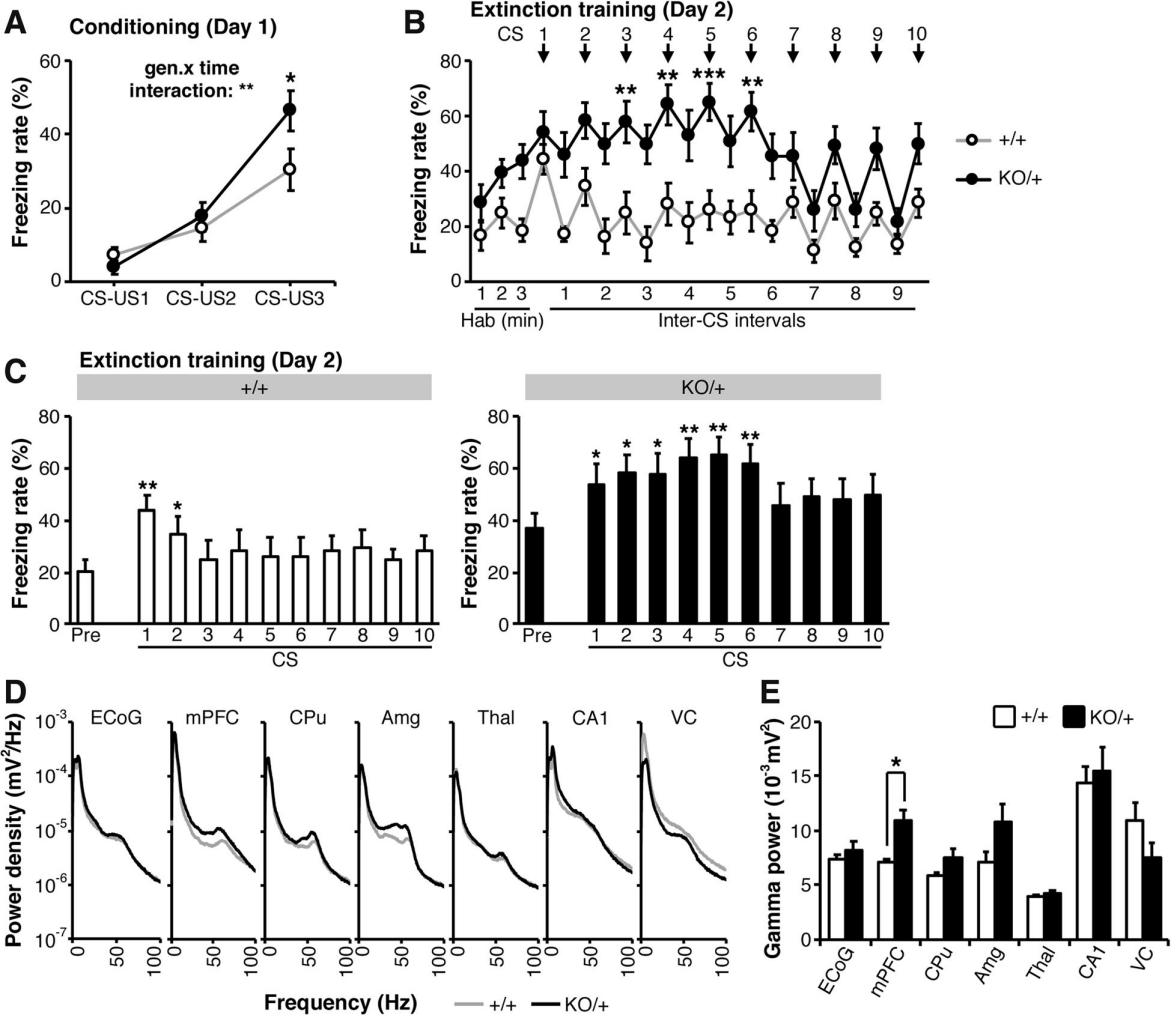
*Scn2a*^KO/+^ mice display enhanced fear memory consolidation, deficient fear extinction, and increased gamma activity in the prefrontal cortex. **a** Mice were conditioned using three CS-US pairs leading to a significant increase in freezing behavior at every consecutive trial. On the third exposition, the freezing percentage was however significantly higher in the *Scn2a*^KO/+^ group (genotype-time interaction: *F*_2,19_ = 8.708, *p* < 0.01). **b** After 24 h, successive exposition to the CS without US rapidly led to a decrease in the freezing percentage of WT mice, whereas freezing ratio remained significantly higher in *Scn2a*^KO/+^ mice and only started to decay after seven consecutive exposition to the tone stimulus (genotype-time interaction: *F*_21,209_ = 1.541, NS; time effect: *F*_21,231_ = 5.096, *p* < 0.001; genotype effect: *F*_1,11_ = 20.580, *p* < 0.001). **c** For further investigation of the fear extinction, the first 3 min of habituation (Pre) were used as a “freezing percentage reference” and freezing during each tone was analyzed in regards to this reference value. In WT (+/+) mice, freezing was significantly increased during the first two tones and decreased to a level that was not significantly different from the habituation period from the third tone. In *Scn2a*^KO/+^ mice, the freezing rate remained significantly higher than the reference level from tone 1 through 6 and decreased to a level that was not significantly different from the habituation period from the tone 7. **d** Power spectrum densities from electrocorticogram and local field potential traces suggested a slight increase in power in the medial prefrontal cortex and the amygdala of *Scn2a*^KO/+^ mice. Traces from other brain regions were comparable between *Scn2a*^KO/+^ and WT samples. **e** The power in the gamma band (30–80 Hz) was significantly increased in the prefrontal cortex of *Scn2a*^KO/+^ mice but not in other regions of the brain. *CS* conditioned stimulus, *US* unconditioned stimulus, *ECoG* electrocorticogram, *mPFC* medial prefrontal cortex, *CPu* caudate putamen, *Amg* basolateral amygdala, *CA1* hippocampal CA1 region, *Thal* ventroposterior thalamus, *VC* visual cortex. Fear memory extinction: WT: *N* = 11, KO: *N* = 12; power spectrum densities: WT: *N* = 4, KO: *N* = 4. Values are expressed as mean ± standard error of the mean. Statistical significance was assessed using two-way repeated measures ANOVA followed by Sidak’s multiple comparison post-hoc test (**a**, **b**) or Student *t* test (**c**–**e**) with significance set at *p < 0.05, **p < 0.01, and ***p < 0.001

Next, we subjected *Scn2a*^KO/+^ mice to different paradigms in the fear conditioning task. Two consecutive auditory cues paired with a foot-shock aversive stimulus resulted in a significant increase in freezing behavior in both *Scn2a*^KO/+^ and WT groups without significant genotype-linked differences (Additional file 2: Figure S10A). Thus, *Scn2a*^KO/+^ mice associated the auditory conditioning stimulus (CS) to the aversive foot-shock stimulus (unconditioned stimulus, US). After 24 h, when placed in the same environment without exposure to either CS or US, *Scn2a*^KO/+^ mice showed a tendency to freeze more than their WT littermates without however reaching the significance level (Additional file 2: Figure S10B). On the following day, mice were exposed to the auditory stimulus (CS) without the aversive US in a new context. At the onset of the auditory cue, we observed a significant increase in the freezing rate in both WT and *Scn2a*^KO/+^ groups (Additional file 2: Figure S10C). The freezing rate was however significantly higher in the *Scn2a*^KO/+^ group for the whole period of the task. As we did not observe differences in freezing behavior in *Scn2a*^KO/+^ mice prior to conditioning (Fig. 5a), this higher freezing on the third day is likely to be a consequence of the fear conditioning rather than a genuine enhanced freezing behavior in these mice. Taken together, these results led us to suspect an enhanced fear conditioning retention in *Scn2a*^KO/+^ mice.

We next investigated the memory extinction ability of *Scn2a*^KO/+^ mice. For that purpose, we used a different set of subject mice in order to prevent any residual effect from a past experience in the fear-conditioning task. The freezing rate increased with the successive exposure to CS-US paired stimuli indicating a successful conditioning in *Scn2a*^KO/+^ and WT mice, and it resulted in a significantly higher freezing rate on the third CS-US pair in *Scn2a*^KO/+^ mice suggesting a stronger conditioning (Fig. 5a). After 24 h, mice were placed in the same environment and exposed to ten consecutive auditory-cues without foot shock. *Scn2a*^KO/+^ mice showed a persistent strong response to successive stimuli compared to their WT littermates and their freezing behavior only started to decay after the 7th exposure to the CS auditory cue (Fig. 5b). In order to assess whether successful fear extinction occurred, we analyzed the freezing rate at each CS presentation using the average freezing rate during the initial three minutes habituation period as reference (Fig. 5c). In WT mice, the freezing rate was significantly higher at CS1 and CS2 before returning to a level not significantly different from the reference. *Scn2a*^KO/+^ mice however showed a freezing rate significantly higher than their reference level from CS1 through CS6 before returning to a level comparable to the reference state. A similar persistent freezing behavior was still observed 48 h after the conditioning phase (Additional file 2: Figure S11). Taken together, these results suggest that the response to fear-conditioning is enhanced in *Scn2a*^KO/+^ mice, whereas the extinction of fear-related memory appears significantly impaired.

### Gamma activity is increased in the prefrontal cortex of *Scn2a*^KO/+^ mice

Because the medial prefrontal cortex and in particular its activity in the gamma band are involved in the consolidation and extinction of fear memories [46] and abnormalities have been reported in the cerebral electric activity in models of SCZ and ASD [47, 48], we electrophysiologically investigated the cerebral cortex of *Scn2a*^KO/+^ mice. Apart from minor epileptiform patterns [33], electrocorticogram (ECoG) recordings did not show significant differences in power spectrum density in transcranial brain surface activity in *Scn2a*^KO/+^ mice during awake periods (Fig. 5d). However, local field potentials (LFPs), providing local activity within various regions of the brain, revealed a significantly higher activity in the gamma-band (30–80 Hz) in the medial prefrontal cortex (mPFC) of *Scn2a*^KO/+^ mice (Fig. 5d-e).

## Discussion

In this study, we completed the first comprehensive behavioral characterization of *Scn2a*^KO/+^ mice. The first striking observation was a novelty-induced hyperactivity (in the sign of longer distance traveled and increased vertical activity) associated with a decrease in anxiety in this model. Hyperactivity is a robust symptom frequently observed in rodent models of SCZ [49–51]. Remarkably, this hyperactivity was recovered by treating *Scn2a*^KO/+^ mice with a potentiator of AMPA receptors and the increased rearing was reproduced by eliminating one of the *Scn2a* alleles specifically in dorsal-telencephalic excitatory neurons. Aside from the “increased dopaminergic signaling” hypothesis in SCZ [49, 52], a second co-existing model involves a deficient glutamatergic signaling focused on the role played by NMDA receptors [50, 51]. Our data are consistent with this alternative proposal and suggest that potentiating AMPA receptor activity is a plausible strategy to rescue hyperactivity.

Recent large-scale studies have identified mutations in *SCN2A* in cases of SCZ resistant to clozapine, an antipsychotic drug acting on dopamine, serotonin, acetylcholine, and noradrenaline receptors commonly used to treat negative symptoms of SCZ [17, 18]. These reports however fail to describe the exact phenotype observed in such patients. In the most commonly used diagnosis screen (DSM-IV), SCZ is identified by the presence of at least two symptoms from a list of positive (hyperactivity, hallucinations, delusions, thought disorders…) and/or negative (affective blunting, reduced expression of emotions, avolition, memory impairment…) behavioral markers combined with abnormal social behavior [53]. Whereas hallucinations and delusions are difficult to investigate in mice, the study of various genetic models helped identify key behavioral markers [49]. In addition to novelty-induced hyperactivity, *Scn2a*^KO/+^ mice displayed an increase in contextual fear memory consolidation and a deficit in locomotor coordination resembling the *sdy* dysbindin mutant mice [54–56]. Though milder than the spectrum of social deficits reported in other SCZ models [49], we observed a decrease in social behavior approach coupled with an experience-dependent social dominance in *Scn2a*^KO/+^ mice. These mice also displayed signs of depression-like behavior in the forced-swim test, a task proposed as a way to detect avolition-like behavior in mice [43], even though further work is required to understand why the tail suspension task showed contradictory results. Startle response and prepulse inhibition, a standard assessment of sensorimotor gating deficient in several models of SCZ [56–58], were however unaffected in *Scn2a* mutant animals.

From the precise case reports of ASD patients with *SCN2A* loss-of-function mutations, the psychomotor delay consistently appears along with primary autistic features: deficits in sociability and communication and repetitive behavior [12, 14–16]. Such deficits are associated with less penetrant phenotypes such as febrile seizures and hyperactivity [12]. Our *Scn2a*^KO/+^ mice recapitulate part of these behavioral phenotypes (locomotor dysfunction, decreased social approach, hyperactivity) but we did not observe communication deficits in juvenile mice nor specific signs of repetitive behavior. Circling behavior or excessive grooming did not accompany the increased rearing behavior in the open field and is thus likely due to the hyperactivity observed in these animals. Moreover, whereas *Scn2a*^KO/+^ mice appeared less sensitive to novelty and elevation-induced anxiety, they were more sensitive to the intense brightness in the light/dark box task.

Overall, *Scn2a*^KO/+^ mice display a small subset of the main behavioral markers characteristic of ASD, together with an ensemble of SCZ-like phenotypes. This observation is consistent with human genetics as mutations in *SCN2A* have been observed in patients of both ASD and SCZ and as there is growing evidence that ASD, SCZ, and ID overlap both in terms of phenotype and candidate genes [17].

Among these overlapping patterns, associative memory and emotion-driven behavior are affected in patients with ASD [59] as well as in persons affected by SCZ [53]. The contextual fear conditioning is the reference task used to decipher these parameters in rodents. In the present study, contextual fear learning was enhanced and the extinction of fear-related memory decreased in *Scn2a*^KO/+^ mice. Similar results have been reported in models of ASD [60] and SCZ [54, 55]. Fear-related behavior relies on a complex network involving amygdala and the medial prefrontal cortex (mPFC) [61, 62]. Whereas the function of this network is difficult to assess in the human brain, several reports pointed to abnormal activity in the gamma band in the prefrontal area of the brain from patients with SCZ [63, 64]. Similarly, the neuronal activity in the gamma band was increased in the mPFC of *Scn2a*^KO/+^ mice. The co-occurrence of a deficit in fear memory extinction and abnormal mPFC activity suggests that a deficit in the mPFC-amygdala circuit might underlie the behavioral phenotype in our model mice. Recent work described more precisely the involvement of the locus coeruleus (LC) noradrenergic system in the control of fear learning and extinction via a modulation of input to the amygdala and mPFC [65]. Further work addressing the LC-amygdala-mPFC in *Scn2a* mutant mice will provide valuable insights into potential mechanisms leading to their behavioral phenotypes.

## Conclusion

*Scn2a*^KO/+^ mice display a set of abnormal phenotypes overlapping patterns that have been observed in model mice of SCZ and ASD. Our data are consistent with the notion that enhancement of glutamatergic transmission restores aspects of some of these phenotypes. This is especially important given the fact that a number of mutations in *SCN2A* were identified in patients with antipsychotic drug-resistant SCZ and that such drugs are actually ineffective in most SCZ patients [17, 18, 66].

## Additional files

Additional file 1: **Table S1.** Animal groups and experimental design. (XLSX 11 kb)

Additional file 2: **Figure S1.** General health and motor function in Scn2a KO mice. **Figure S2.** *Scn2a*^KO/+^ mice show hyperactivity and low anxiety during the first 60 min of exploration in a new environment. **Figure S3.** Exploratory activity is not significantly affected in the elevated plus maze task in Emx1- and Vgat-Cre conditional *Scn2a* knockout mice. **Figure S4.** Treatment by AMPA receptor modulator CX516 shows a maximal effect at 40 mg/kg for spontaneous exploratory behavior assessment. **Figure S5.** Treatment with the AMPA receptor modulator CX516 did not affect the decrease in anxiety-related behavior of *Scn2a*^KO/+^ in the elevated-plus maze. **Figure S6.** CX516 injection does not affect significantly locomotor coordination performance. **Figure S7.** Habituation and activity in the 3-chambers task were comparable between *Scn2a*^KO/+^ and WT mice. **Figure S8.** Isolation-induced ultrasonic vocalizations are conserved in *Scn2a*^KO/+^ pups at P6. **Figure S9.** *Scn2a*^KO/+^ mice display despair behavior in the forced-swim task but conserved startle and pre-pulse inhibition response. **Figure S10.** Freezing behavior is increased in *Scn2a*^KO/+^ mice three days post-conditioning. **Figure S11.** Fear memory induced freezing remains abnormally elevated in *Scn2a*^KO/+^ mice 48 h after conditioning. (PDF 4068 kb)

Additional file 3: Additional material and methods for experiments displayed in the Additional file 2. Includes protocols for ultrasonic vocalizations, motor and sensory functions, forced-swim and tail suspension tests, and startle response and prepulse inhibition. (DOCX 16 kb)

## Abbreviations

ASD: Autism spectrum disorder
BFNIS: Benign familial neonatal-infantile seizures
CS: Conditioned stimulus
EE: Epileptic encephalopathies
FST: Forced swim test
GEFS+: Generalized epilepsy with febrile seizures plus
ID: Intellectual disability
LFP: Local field potential
MSN: Medium spiny neurons
PPI: Prepulse inhibition
SCZ: Schizophrenia
TST: Tail suspension test
US: Unconditioned stimulus
VGSCs: Voltage-gated sodium channels
