# Supplementary material for: Scn2a haploinsufficient mice display a spectrum of phenotypes affecting anxiety, sociability, memory flexibility and ampakine CX516 rescues their hyperactivity
Source: Mol Autism. 2019 Mar 28;10:15. doi: 10.1186/s13229-019-0265-5 (PMC6437867; doi:10.1186/s13229-019-0265-5)
Supplement: Supplementary file 2 — Figure S1. General health and motor function in Scn2a KO mice. Figure S2. Scn2aKO/+ mice show hyperactivity and low anxiety during the first 60 min of exploration in a new environment. Figure S3. Exploratory activity is not significantly affected in the elevated plus maze task in Emx1- and Vgat-Cre conditional Scn2a knockout mice. Figure S4. Treatment by AMPA receptor modulator CX516 shows a maximal effect at 40 mg/kg for spontaneous exploratory behavior assessment. Figure S5. Treatment with the AMPA receptor modulator CX516 did not affect the decrease in anxiety-related behavior of Scn2aKO/+ in the elevated-plus maze. Figure S6. CX516 injection does not affect significantly locomotor coordination performance. Figure S7. Habituation and activity in the 3-chambers task were comparable between Scn2aKO/+ and WT mice. Figure S8. Isolation-induced ultrasonic vocalizations are conserved in Scn2aKO/+ pups at P6. Figure S9. Scn2aKO/+ mice display despair behavior in the forced-swim task but conserved startle and pre-pulse inhibition response. Figure S10. Freezing behavior is increased in Scn2aKO/+ mice three days post-conditioning. Figure S11. Fear memory induced freezing remains abnormally elevated in Scn2aKO/+ mice 48 h after conditioning. (PDF 4068 kb) [file 13229_2019_265_MOESM2_ESM.pdf]

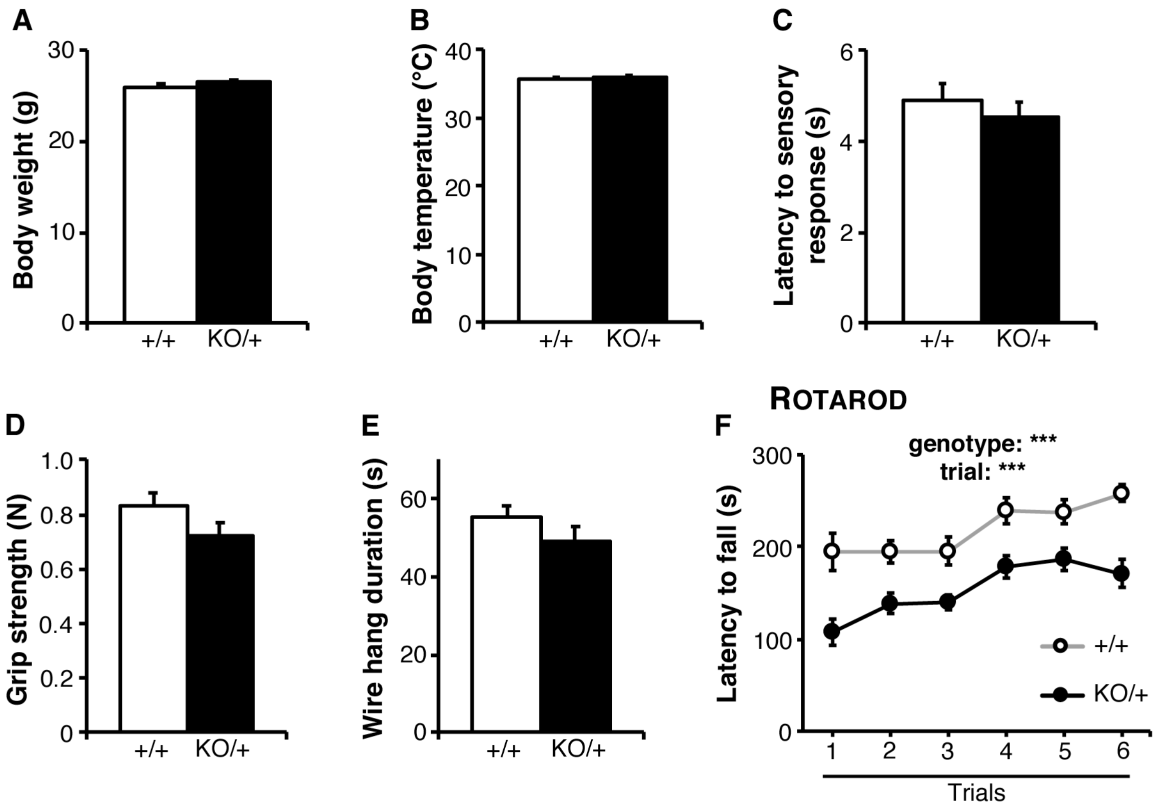

Figure S1. General health and motor function in *Scn2a* KO mice. At 11-weeks-old, body weight (**A**) and temperature (**B**) were not significantly different between *Scn2a*$^{KO/+}$ mice and their WT littermates. In the hot plate test (**C**) the latency to sensory response was comparable between the two groups. The grip strength (**D**) and the performance in the wire hang test (**E**) in *Scn2a*$^{KO/+}$ mice was comparable to that of their littermates. However, *Scn2a*$^{KO/+}$ mice performed significantly worse in the rotarod task (**F**) as their latency to fall off the rod was significantly shorter than their WT counterparts (genotype-trial interaction: $F_{5,95} = 1.095$, *NS*; trial effect: $F_{5,95} = 16.220$, $p < 0.001$; genotype effect: $F_{1,19} = 32.440$, $p < 0.001$). WT: N = 20, KO: N = 20. Values are expressed as mean ± standard error of the mean. Statistical significance was assessed using one-way ANOVA (A-E) or two-way repeated measures ANOVA (F) with significance set at (*) $p < 0.05$, (**) $p < 0.01$ and (***) $p < 0.001$.

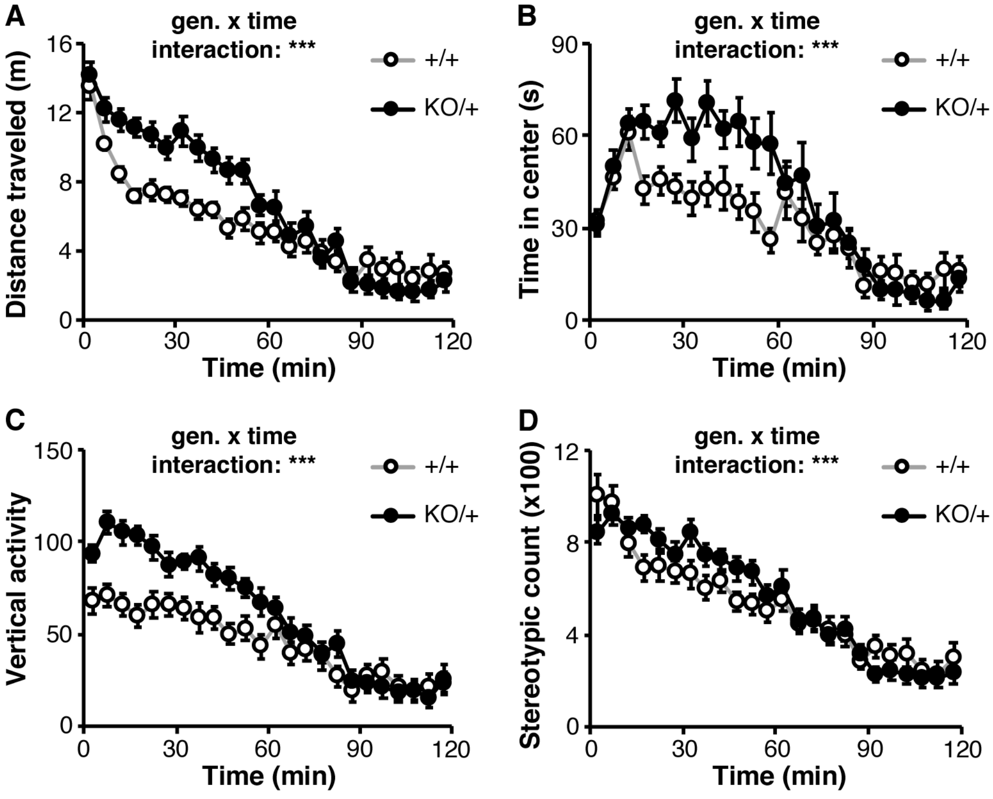

Figure S2. *Scn2a*$^{KO/+}$ mice show hyperactivity and low anxiety during the first 60 min of exploration in a new environment. (**A**) When *Scn2a*$^{KO/+}$ mice were left to explore freely an open field over a 2 hours period, they traveled a significantly longer distance during the initial 60 minutes (genotype-time interaction: $F_{23,437} = 5.832$, $p < 0.001$; post-hoc time points 3 to 11: $p < 0.01$). They also spent significantly more time at the center of the open field (**B**, genotype-time interaction: $F_{23,437} = 3.725$, $p < 0.001$; post-hoc time points 4, 6, 8, 10-12: $p < 0.05$) and reared significantly more (**C**, genotype-time interaction: $F_{23,437} = 5.698$, $p < 0.001$; post-hoc time points 1 to 12: $p < 0.05$) than their WT littermates. The total number of stereotypic counts (**D**) was however overall not significantly different between the groups (genotype-time interaction: $F_{23,437} = 3.043$, $p < 0.001$; post-hoc time points 4 and 7: $p < 0.05$). WT: N = 20, KO: N = 20. Values are expressed as mean ± standard error of the mean. Statistical significance was assessed using two-way repeated measures ANOVA with significance set at $p < 0.05$.

ELEVATED PLUS MAZE

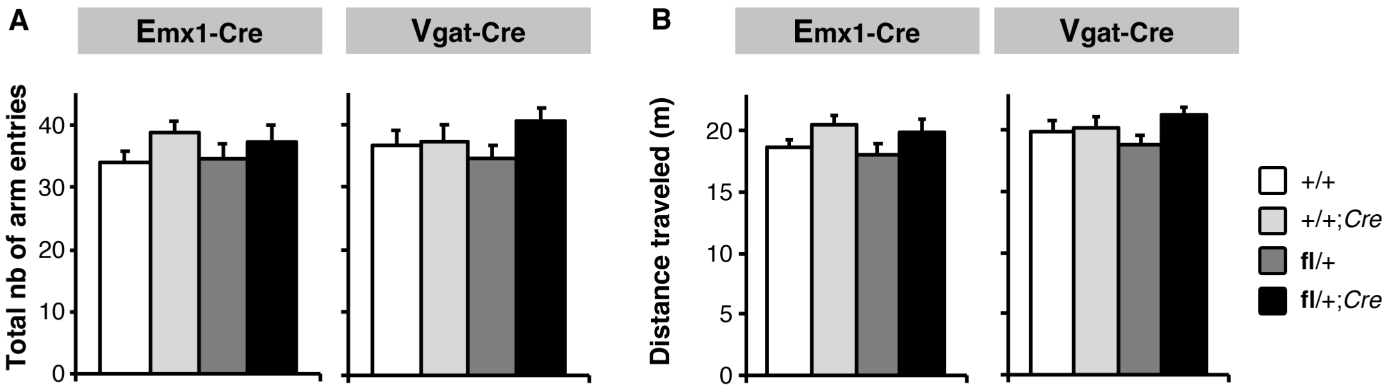

Figure S3. Exploratory activity is not significantly affected in the elevated plus maze task in *Emx1*- and *Vgat*-Cre conditional *Scn2a* knockout mice. (**A**) The total number of arm entries in the elevated plus maze task was not significantly affected in mice carrying a heterozygous deletion of *Scn2a* in dorsal-telencephalic excitatory neurons (*Emx1*-Cre) nor in mice with a heterozygous deletion of *Scn2a* in inhibitory neurons (*Vgat*-Cre)(*Emx1*-Cre: $F_{3,53}$ = 1.036, *NS*; *Vgat*-Cre: $F_{3,46}$ = 1.331, *NS*). (**B**) No significant differences were observed in the total traveled distance (*Emx1*-Cre: $F_{3,53}$ = 1.595 *NS*; *Vgat*-Cre: $F_{3,46}$ = 1.554, *NS*). Values are expressed as mean ± standard error of the mean. Statistical significance was assessed using one-way ANOVA with significance set at $p < 0.05$.

OPEN FIELD

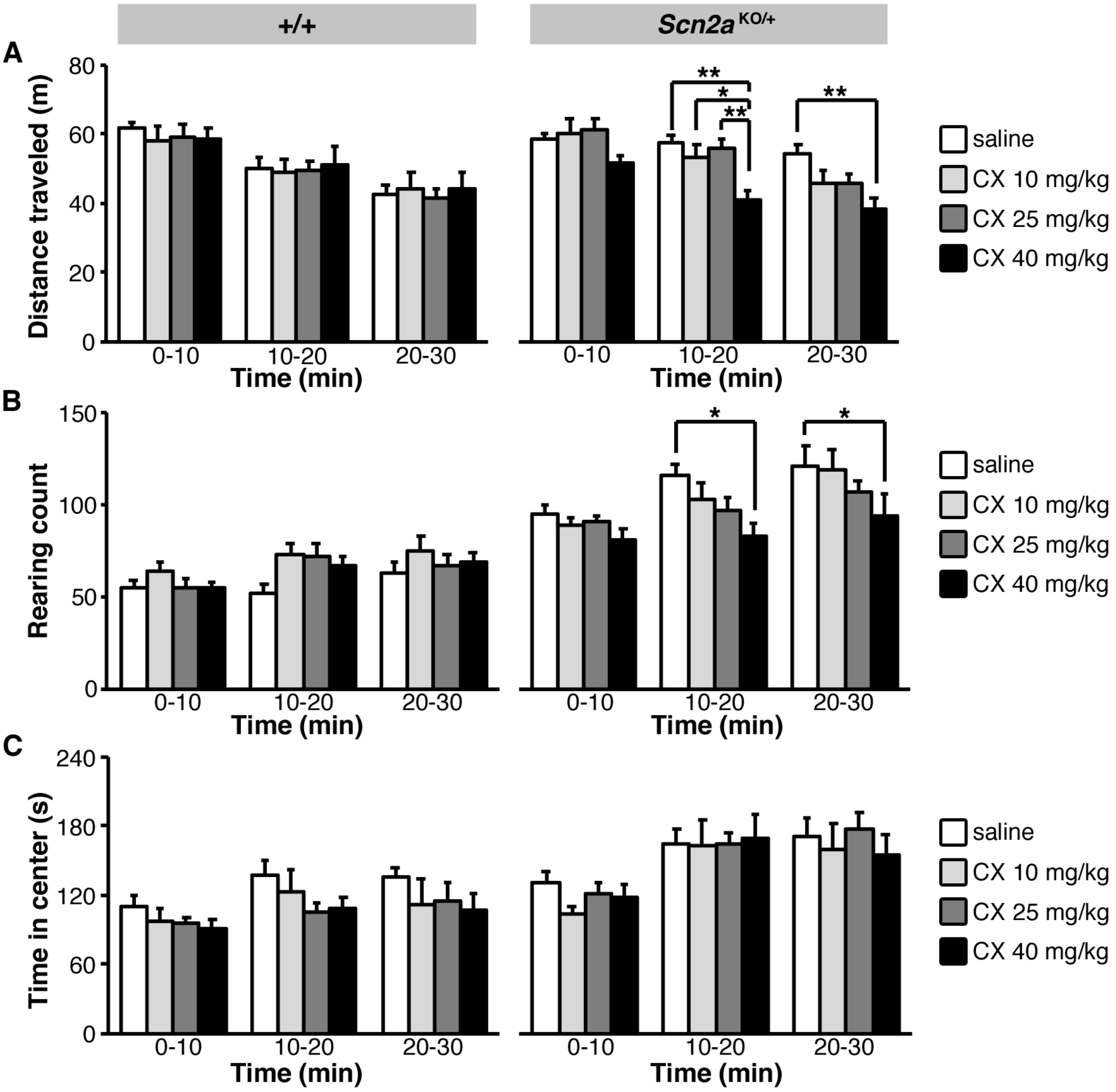

Figure S4. Treatment by AMPA receptor modulator CX516 shows a maximal effect at 40 mg/kg for spontaneous exploratory behavior assessment. CX516, a specific AMPA receptor positive allosteric modulator, was intraperitoneally administered 10 minutes before submitting WT (+/+) and *Scn2a*$^{KO/+}$ mice to the open field task. (**A**) The distance traveled by WT mice decreased over time without a significant effect of CX516 injections at the 3 doses tested (time-drug dose interaction: $F_{6,80} = 0.562$, *NS*; time effect: $F_{2,80} = 173.290$, $p < 0.001$; drug dose effect: $F_{3,40} = 0.044$, *NS*). In *Scn2a*$^{KO/+}$ mice there a CX516 injections led to a significant decrease in traveled distance in the second and third blocks of 10 minutes, with the strongest effect at 40 mg/kg (time-drug dose interaction: $F_{6,80} = 2.426$, $p < 0.05$). (**B**) The rearing count in the WT group showed a slight but significant increase over time without a significant effect of CX516 (time-drug dose interaction: $F_{6,80} = 1.075$, *NS*; time effect: $F_{2,80} = 6.859$, $p < 0.01$; drug dose effect: $F_{3,40} = 1.490$, *NS*). *Scn2a*$^{KO/+}$ mice reared significantly more during the second and third blocks of 10 minutes and CX516 injections suppressed this behavior in a dose dependent manner with a maximum effect at 40 mg/kg (time-drug dose interaction: $F_{6,80} = 1.765$, *NS*; time effect: $F_{2,80} = 15.410$, $p < 0.001$; drug dose effect: $F_{3,40} = 3.866$, $p < 0.05$). (**C**) Over the course of the test, WT mice gradually spent more time in the center and CX516 did not affect significantly this behavior (time-drug dose interaction: $F_{6,80} = 0.408$, *NS*; time effect: $F_{2,80} = 6.626$, $p < 0.01$; drug dose effect: $F_{3,40} = 1.074$, *NS*). In a similar way, the time spent in the center in the *Scn2a*$^{KO/+}$ group increased over time and was not affected by CX516 at the three doses tested (time-drug dose interaction: $F_{6,80} = 0.487$, *NS*; time effect: $F_{2,80} = 23.330$, $p < 0.001$; drug dose effect: $F_{3,40} = 0.271$, *NS*). The test was conducted using N = 11 mice per genotype and per condition (saline, CX516 at 10, 25 or 40 mg/kg). Values are expressed as mean ± standard error of the mean. Statistical significance was assessed using two-way repeated measures ANOVA for treatment and time separately in each group of genotype, followed by Tukey's post-hoc test with significance set at *(*)* $p < 0.05$ and *(**)* $p < 0.01$.

ELEVATED PLUS MAZE

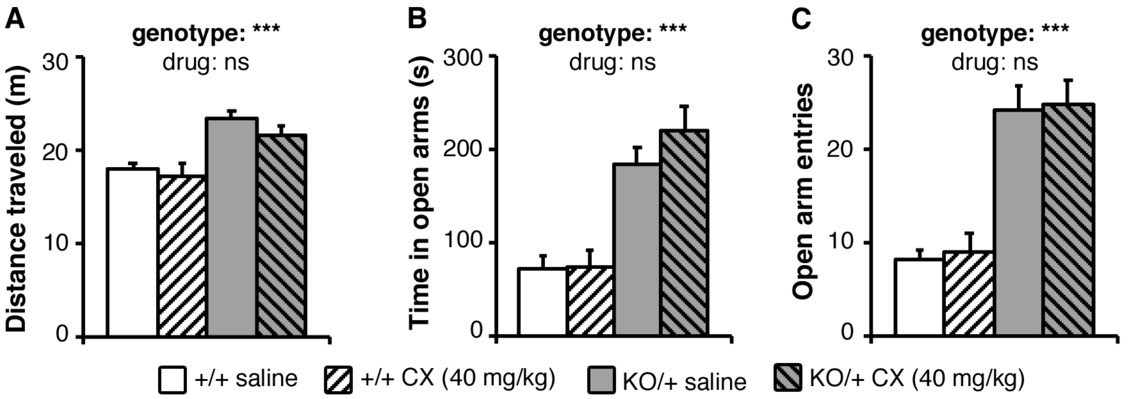

Figure S5. Treatment with the AMPA receptor modulator CX516 did not affect the decrease in anxiety-related behavior of *Scn2a*$^{KO/+}$ in the elevated-plus maze. (**A**) Saline injected *Scn2a*$^{KO/+}$ mice (KO/+) traveled longer distances in the elevated-plus maze than their WT littermates (+/+) and treatment with CX516 at 40 mg/kg did not produce a significant effect (genotype-treatment interaction: $F_{1,43} = 0.295$, *NS*; treatment effect: $F_{1,21} = 2.108$, *NS*; genotype effect: $F_{1,21} = 28.314$, $p < 0.001$). (**B**) *Scn2a*$^{KO/+}$ mice spent significantly more time in the open arms and this was not affected by CX516 injection (genotype-treatment interaction: $F_{1,43} = 0.737$, *NS*; treatment effect: $F_{1,21} = 0.924$, *NS*; genotype effect: $F_{1,21} = 43.933$, $p < 0.001$). (**C**) The number of entries in the open arms of the track was also increased in the *Scn2a*$^{KO/+}$ group and CX516 did not affect significantly this phenotype (genotype-treatment interaction: $F_{1,43} = 0.001$, *NS*; treatment effect: $F_{1,21} = 0.122$, *NS*; genotype effect: $F_{1,21} = 58.400$, $p < 0.001$). N = 11 for each group of genotype and each condition. Values are expressed as mean ± standard error of the mean. Statistical significance was assessed using two-way ANOVA for genotype and treatment effect with significance set at (*) $p < 0.05$ and (***) $p < 0.001$.

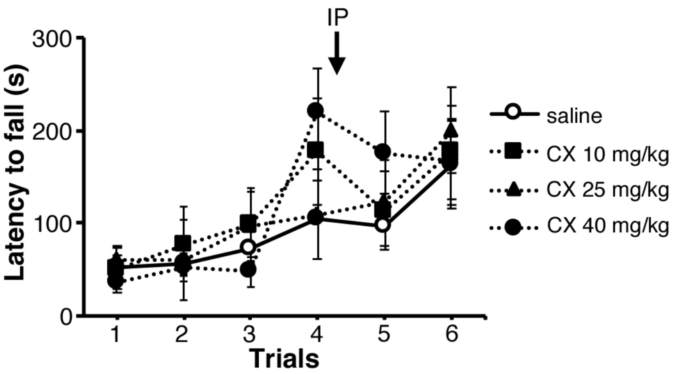

Figure S6. CX516 injection does not affect significantly locomotor coordination performance. Injection of CX516 in WT mice did not affect significantly the performance in the rotarod task at the different doses tested (trial-drug dose interaction: $F_{15,80} = 0.899$, *NS*; trial effect: $F_{5,80} = 10.690$, $p < 0.001$; drug dose effect: $F_{3,16} = 0.260$, *NS*). N = 5 per condition. Values are expressed as mean ± standard error of the mean. Statistical significance was assessed using two-way repeated measures ANOVA with significance set at $p < 0.05$.

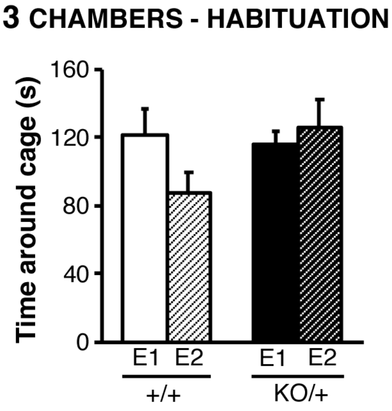

Figure S7. Habituation and activity in the 3-chambers task were comparable between *Scn2a*$^{KO/+}$ and WT mice. During the habituation to the maze, we did not observe a significant preference for a given side in either group. E1: Empty side 1; E2: Empty side 2. WT: N = 23, KO: N = 29. Values are expressed as mean ± standard error of the mean. Statistical significance was assessed using Student t-test with significance set at $p < 0.05$.

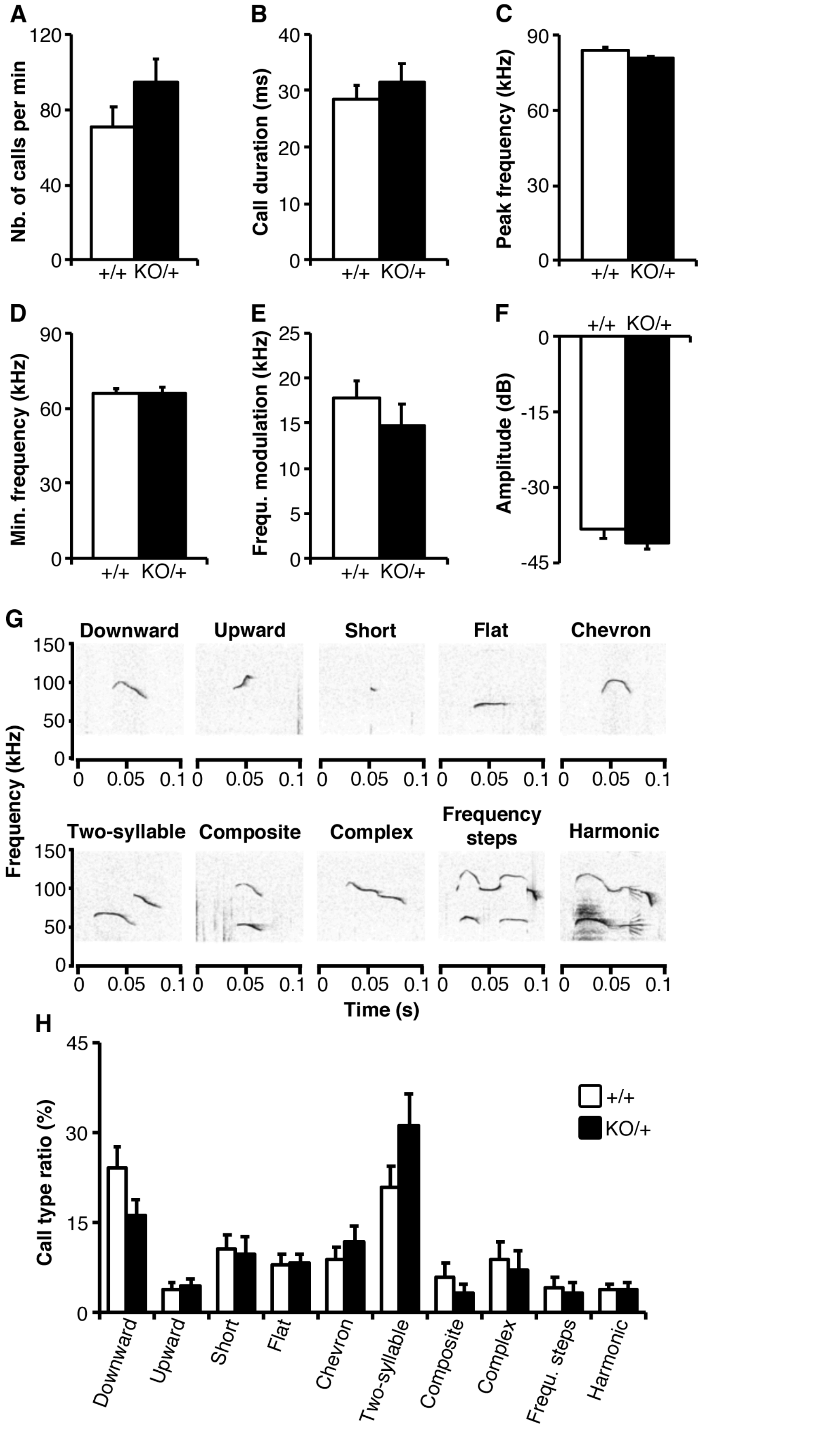

Figure S8. Isolation-induced ultrasonic vocalizations are conserved in *Scn2a*$^{KO/+}$ pups at P6. (A-F) Isolation-induced ultrasonic vocalizations recorded at postnatal 6 days were analyzed quantitatively and qualitatively using frequency, duration and amplitude parameters. *Scn2a*$^{KO/+}$ mice produced a number of calls (**A**) comparable to their WT littermates. The mean duration (**B**), peak frequency (**C**), minimum frequency (**D**), frequency modulation (**E**) and amplitude (**F**) were not significantly different between *Scn2a*$^{KO/+}$ and WT pups. (**G**) Calls were classified into the 10 main categories commonly observed in C57BL/6J pups. (**H**) The proportion of calls per category did not differ significantly between the two groups, even for downward and two-syllable calls that seemed decreased and increased respectively without reaching the significance level ($p = 0.110$ and $p = 0.122$ respectively). WT: N = 8, KO: N = 8. Values are expressed as mean ± standard error of the mean. Statistical significance was assessed using Student t-test with significance set at $p < 0.05$.

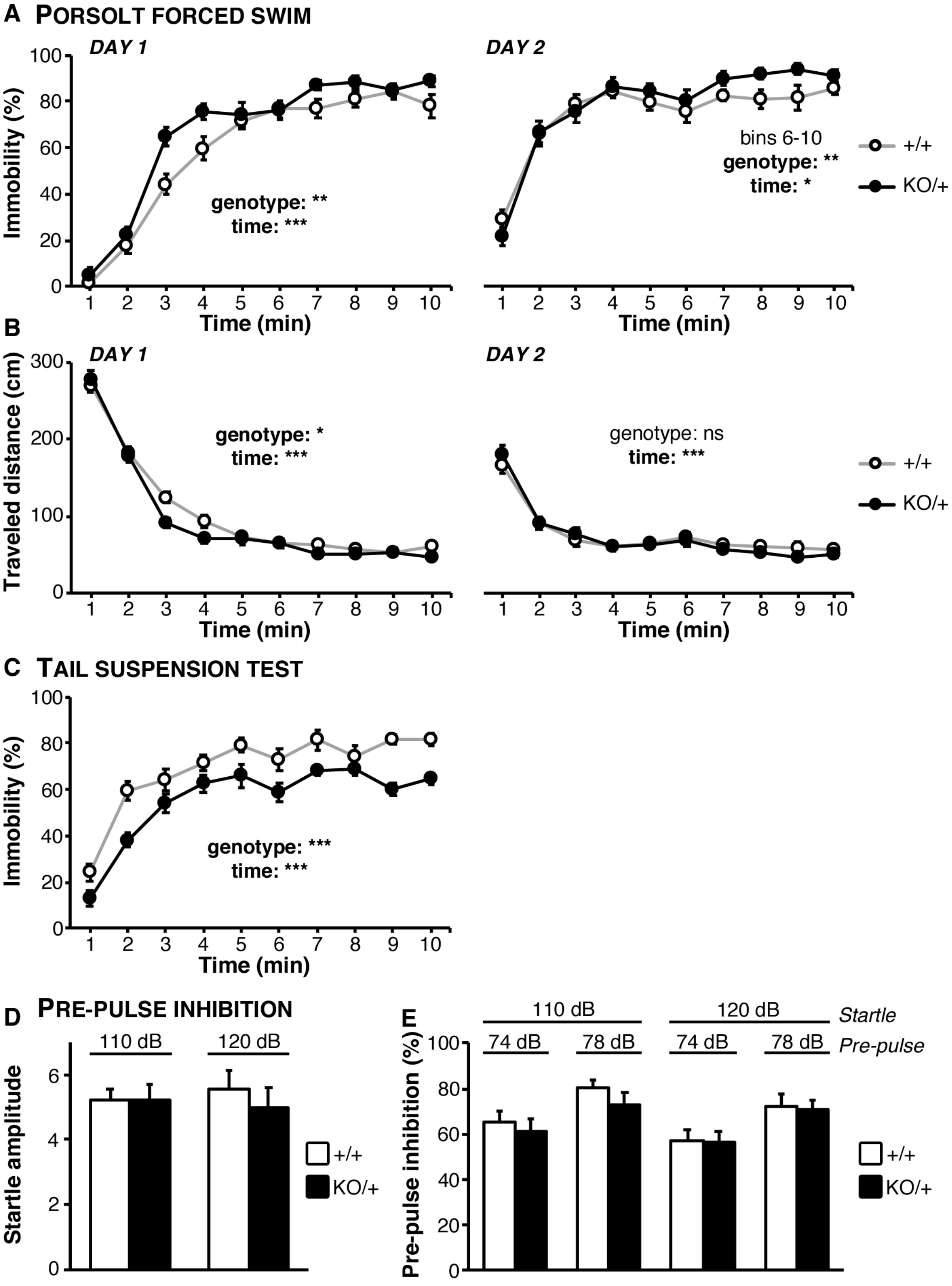

Figure S9. *Scn2a*$^{KO/+}$ mice display despair behavior in the forced-swim task but conserved startle and pre-pulse inhibition response. (**A**) In the Porsolt forced swim test, *Scn2a*$^{KO/+}$ mice were significantly more immobile than their WT littermates on day 1 and in the second half of day 2 (Day1: genotype-time interaction: $F_{9,171}$ = 2.094, *NS*, time effect: $F_{9,171}$ = 132.7, $p < 0.001$; genotype effect: $F_{1,19}$ = 13.06, $p < 0.01$; Day2: genotype-time interaction: $F_{9,171}$ = 1.661, *NS*, time effect: $F_{9,171}$ = 48.420, $p < 0.001$; genotype effect: $F_{1,19}$ = 3.102, $p > 0.01$; Day2: bins 6-10: genotype-time interaction: $F_{4,76}$ = 0.533, *NS*, time effect: $F_{4,76}$ = 3.077, $p < 0.05$; genotype effect: $F_{1,19}$ = 8.572, $p < 0.01$). (**B**) Conversely, *Scn2a*$^{KO/+}$ mice traveled shorter distances on the first day tested, but not on the second day (Day 1: genotype-time interaction: $F_{9,171}$ = 1.708, *NS*, time effect: $F_{9,171}$ = 234.4, $p < 0.001$; genotype effect: $F_{1,19}$ = 4.625, $p < 0.05$; Day 2: genotype-time interaction: $F_{9,171}$ = 0.957, *NS*, time effect: $F_{9,171}$ = 58.160, $p < 0.001$; genotype effect: $F_{1,19}$ = 0.025, *NS*). (**C**) In the tail suspension test, *Scn2a*$^{KO/+}$ mice were significantly less immobile than their WT littermates (genotype-time interaction: $F_{9,171}$ = 0.740, *NS*, time effect: $F_{9,171}$ = 42.560, $p < 0.001$; genotype effect: $F_{1,19}$ = 28.290, $p < 0.001$). (**D**) No significant differences were observed between the *Scn2a*$^{KO/+}$ and WT groups in startle response for the two sound levels used (110 dB and 120 dB). (**E**) The response to pre-pulse inhibition was also comparable between both groups for all the stimulation thresholds tested. WT: N = 20, KO: N = 20. Values are expressed as mean ± standard error of the mean. Statistical significance was assessed using two-way repeated measures ANOVA (A-C) or two-way ANOVA (D,E) with significance set at $p < 0.05$.

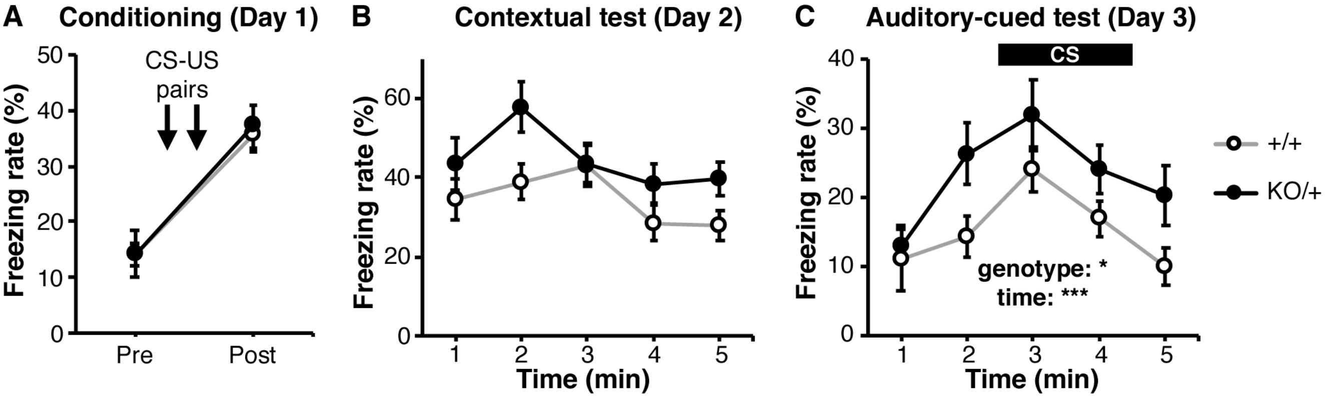

Figure S10. Freezing behavior is increased in *Scn2a*$^{KO/+}$ mice three days post-conditioning. Mice were tested in a fear conditioning, contextual and auditory cued testing paradigm. (**A**) In the conditioning phase of the fear conditioning test, the freezing behavior was significantly increased by two pairs of conditioned (CS, tone) and unconditioned stimuli (US, foot shock) in *Scn2a*$^{KO/+}$ and WT mice without significant genotype effect (genotype-time interaction: $F_{1,12} = 0.075$, *NS*; time effect: $F_{1,12} = 48.570$, $p < 0.001$; genotype effect: $F_{1,12} = 0.039$, *NS*). (**B**) After 24 hours, when mice were placed back in the same environment without CS or US, *Scn2a*$^{KO/+}$ mice tended to freeze more than their WT littermates even though this did not reach the significance level (genotype-time interaction: $F_{4,48} = 1.644$, *NS*; time effect: $F_{4,48} = 48.570$, $p < 0.01$; genotype effect: $F_{1,12} = 4.574$, $p = 0.086$). (**C**) In the auditory cued test, 24 hours later, mice were placed in a completely different environment and exposed to one CS without US. The freezing percentage was significantly higher in the *Scn2a*$^{KO/+}$ group (genotype-time interaction: $F_{4,48} = 0.639$, *NS*; time effect: $F_{4,48} = 9.743$, $p < 0.001$; genotype effect: $F_{1,12} = 5.399$, $p < 0.05$). WT: N = 13, KO: N = 13. Values are expressed as mean ± standard error of the mean. Statistical significance was assessed using two-way repeated measures ANOVA with significance set at *(*)* $p < 0.05$.

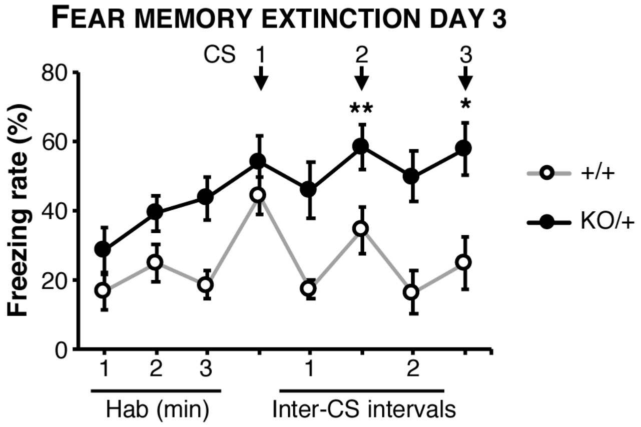

Figure S11. Fear memory induced freezing remains abnormally elevated in *Scn2a*$^{KO/+}$ mice 48 hours after conditioning. Mice were trained for fear conditioning by exposition to tone (CS: conditioned stimulus) paired to electric foot-shocks (US: unconditioned stimulus). Forty-eight hours post-conditioning, *Scn2a*$^{KO/+}$ mice showed a significantly stronger freezing behavior when exposed to three consecutive pairs of CS without US. WT: N = 11, KO: N = 12. Values are expressed as mean ± standard error of the mean. Statistical significance was assessed using two-way repeated measures ANOVA followed by Sidak's multiple comparison post-hoc test with significance set at (*) $p < 0.05$ and (**) $p < 0.01$.
