# Supplementary material for: Scn2a haploinsufficient mice display a spectrum of phenotypes affecting anxiety, sociability, memory flexibility and ampakine CX516 rescues their hyperactivity
Source: Mol Autism. 2019 Mar 28;10:15. doi: 10.1186/s13229-019-0265-5 (PMC6437867; doi:10.1186/s13229-019-0265-5)
Supplement: Supplementary file 3 — Additional material and methods for experiments displayed in the Additional file 2. Includes protocols for ultrasonic vocalizations, motor and sensory functions, forced-swim and tail suspension tests, and startle response and prepulse inhibition. (DOCX 16 kb) [file 13229_2019_265_MOESM3_ESM.docx]

# Supplementary Methods

## Ultrasonic vocalizations (USV)

Isolation-induced USV were recorded from pups separated from their mother for 5 min at post-natal day 6. Pups were individually removed from the nest and placed in a plastic cylindrical container on top of fresh nesting material. The container was placed in an acoustic attenuation box and a microphone adjusted 10 cm above the pup. Vocalizations were recorded for 5 minutes at a 250,000 Hz sampling rate in 16-bit format using Avisoft Recorder (version 4.2.8; Avisoft Bioacoustics, Berlin, Germany). Recorded files were processed using Avisoft SAS Lab Pro (version 5.1; Avisoft Bioacoustics, Berlin, Germany). A fast Fourier transform was applied (512 FFT length, 100% frame size, Hamming window and 75% time window overlap) and spectrograms generated with a 0.512 ms time resolution and 488 Hz frequency resolution. A high-pass filter set at 30 kHz was applied to reduce background noise outside of the USV relevant frequency band. USV calls were automatically detected using a double threshold-based algorithm (amplitude: -40dB, hold time: 10 ms). An experimenter blind to the genotype of the pups verified the accuracy of the detected calls and, when necessary, manually added or corrected missed and incorrectly detected calls. Call duration, peak amplitude, peak frequency, minimum frequency and frequency modulation were averaged for every mouse. Calls were classified into ten categories commonly observed in C57BL/6 pups (Scattoni et al., 2008; Raveau et al., 2018).

## Motor function

Motor function tests, including grip strength, wire hang and rotarod tasks, were conducted as previously described (Nakao et al., 2015). A wire hang test apparatus, commonly used to assess grip strength, is a wire mesh grid (10 × 10 cm) placed over of a box (21.5 × 22 × 23 cm; O’Hara & Co., Tokyo, Japan). A trigger-activated inversion of the grid causes mice to grip the wire. The latency to fall was recorded with a 60 sec cut-off time. A grip strength meter (O’Hara & Co., Tokyo, Japan) was used to assess forelimb grip strength. Mice were lifted and held by the tail so that their forepaws could grasp the wire grid. They were then gently pulled horizontally backward by the tail until they released the grid. The peak grip-force applied by the forelimbs, expressed in Newton (N), was recorded. Each mouse was tested three times, and the highest value was used for statistical analysis. Motor coordination and balance were tested using the accelerating rotarod paradigm (UGO Basile Accelerating Rotarod, Varese, Italy or Rota-rod treadmill, MK-610A, Muromachi Kikai Co., Ltd, Tokyo, Japan). Mice were placed on a 3 cm diameter polyvinyl chloride-coated rod linearly accelerating from 4 to 40 rpm over 5 min and the latency to fall from the rod was recorded.

## Sensory function: hot plate test

The hot plate test was conducted to evaluate the sensory sensitivity in response to a painful stimulus. Mice were placed on a 55.0 ± 0.3°C hot plate (Columbus Instruments, Columbus, OH), and the latency to the first paw response was recorded with a 15-sec cut-off time. The hind-paw response was defined as either a foot shake or paw lick.

## Porsolt forced swim

Four Plexiglas cylinders (20 cm height × 10 cm diameter) were filled with 7.5 cm of water at room temperature. During two consecutive days, mice were placed in the cylinders and their behavior recorded for 10 min. Images were captured at two frames per second and animal’s body automatically detected. For each pair of successive frames, the mouse was considered as ‘immobile’ if the body’s area overlapped more than the threshold. It was otherwise considered as ‘moving’. Immobility events lasting for < 2 s (i.e. less than three consecutive frames) were not included. Data acquisition and analysis were performed automatically the ImageJ-based ImageTS software (designed by Tsuyoshi Miyakawa, available through O’HARA&Co., Tokyo, Japan).

## Tail suspension

Mice were suspended above the floor of a white plastic chamber (31 × 41 × 41 cm; O’Hara & Co., Tokyo, Japan) in a visually isolated area using adhesive tape placed 1 cm from the base of the tail. The animal’s behavior was recorded for 10 min (acquisition at two frames per second). Immobility events were assessed as described above for the Porsolt forced swim test. Data acquisition and analysis were performed automatically the ImageJ-based ImageTS software (designed by Tsuyoshi Miyakawa, available through O’HARA&Co., Tokyo, Japan).

## Startle response/prepulse inhibition

The startle response/prepulse inhibition test was conducted as previously described (Nakao et al., 2015). Mice were placed in a Plexiglas cylinder with a 70 dB white noise background and left for 10 min of habituation. An auditory stimulus (40 ms; 110 or 120 dB) was then applied, the behavioral response recorded for 400 ms from the onset of the stimulus and the peak startle response was measured. During the pre-pulse inhibition phase, this startle stimulus was preceded by a pre-pulse sound (20 ms; 74 or 78 dB). Each test block was thus composed of two startles (110 and 120 dB) and four pre-pulse events (prepulse: 74 dB + startle 110 and 120 dB; prepulse 78 dB + startle 110 and 120 dB). Each mouse experienced six blocks. The average inter-trial interval was 15 s (range: 10–20 s). Startle response and pre-pulse inhibition were automatically detected using the manufacturer’s software (O’Hara & Co., Tokyo, Japan).

***Reference***

Nakao A, Miki T, Shoji H, Nishi M, Takeshima H, Miyakawa T et al. Comprehensive behavioral analysis of voltage-gated calcium channel beta-anchoring and -regulatory protein knockout mice. *Front Behav Neurosci* 2015; **9**, 141.
